# High melanosome diversity exhibits weak correlation with color and environmental variables in the early evolution of therian mammals

**DOI:** 10.1126/sciadv.adw8707

**Published:** 2025-10-24

**Authors:** Xin Li, Shundong Bi, Julia A. Clarke, Zhongqiu Li, Xichao Zhu, Quansheng Liu, Yajun Peng, Lingling Zhao, Zhiheng Li, Yanhong Pan

**Affiliations:** ^1^ State Key Laboratory for Mineral Deposits Research, School of Earth Sciences and Engineering, Centre for Research and Education on Biological Evolution and Environment and Frontiers Science, Center for Critical Earth Material Cycling, Nanjing University, Jiangsu 210023, China.; ^2^ Centre for Vertebrate Evolutionary Biology, School of Life Sciences, Yunnan University, Kunming 650091, China.; ^3^ Department of Anatomy, Howard University College of Medicine, Washington, DC 20059, USA.; ^4^ Department of Earth and Planetary Sciences, Jackson School of Geosciences, The University of Texas, TX 78712, USA.; ^5^ Lab of Animal Behavior and Conservation, School of Life Sciences, Nanjing University, Nanjing 210023, China.; ^6^ National Animal Collection Resource Center, Institute of Zoology, Chinese Academy of Sciences, Beijing 100101, China.; ^7^ Guangdong Key Laboratory of Animal Conservation and Resource Utilization, Guangdong Public Laboratory of Wild Animal Conservation and Utilization, Guangdong Institute of Applied Biological Resources, Guangzhou 510260, China.; ^8^ Zhejiang Museum of Natural History, Hangzhou 310014, China.; ^9^ Hongshan Forest Zoo, Nanjing 210028, China.; ^10^ Key Laboratory of Vertebrate Evolution and Human Origins, Institute of Vertebrate Paleontology and Paleoanthropology, Chinese Academy of Sciences, Beijing 100044, China.

## Abstract

Melanosome (melanin-containing organelles) geometries sampled from fossil Jurassic fur have recently indicated uniformly dark colors consistent with a proposed nocturnal bottleneck early in the mammal lineage. Here, we use a distinct dataset of ~8000 melanosomes from 60 species (11 mammalian orders) including a Jurassic species to ask more generally what color or environmental variables may explain melanosome geometry in mammals. We confirm support for limited melanosome variation in Jurassic mammals. Crown therian mammalian melanosome diversity as extreme as that in birds is recovered within placental lineages. More environmental variables substantially explain aspects of melanosome geometry than do color variables, but the explanatory power of both sets of variables is limited. Prediction of color from melanosomes in fossil mammals merits caution, given observed lineage-specific trends. Potentially comparatively weak selection on the melanosome geometry-color relationship in mammals or correlations with other life history variables may further explain the evolution of these geometries.

## Teaser

Melanosome geometry in Jurassic and therian mammals reflected a limited color/environment link unlike that in dinosaurs.

## INTRODUCTION

Coloration in vertebrates serves diverse functions such as aposematism, crypsis, and communication; it shows complex variation in relation to the environment, life history, and ecology generally (*1*, *2*). In amniotes, melanin (eumelanin and pheomelanin) is one of the most broadly expressed pigments, determining many of the yellow, brown, red, and black colors of skin, feathers, and hair (*3*–*6*). The geometries of pigment-containing organelles, melanosomes, have been shown to correlate with color in some amniote taxa, but not others (*7*–*9*). The diversity of melanosome geometries in birds reflects variation both in melanin-based colors as well as their role in generating structural iridescent colors (*9*–*11*). In view of its high fossilization potential (*12*, *13*), melanosome geometry has been used to infer colors in various extinct taxa (*14*, *15*), such as dinosaurs (*8*, *9*, *16*, *17*) including birds (*7*, *16*), pterosaurs (*18*), and, recently, mammals (*19*).

Extant mammals show a wide range of melanosome geometries, comparable to those in birds and feathered dinosaurs and more diverse than in other reptiles (*20*). However, in contrast to feather coloration, mammalian pelage tends to be prominently less varied and duller (*21*). A key recent study reconstructed Mesozoic mammalian pelage color in multiple fossils outside crown Theria as showing a similar, uniform black hue (*19*). The observed limited diversity in pelage color was hypothesized to be related to nocturnal or crepuscular activity patterns proposed for most early mammals (*19*, *22*, *23*), as well as reduced sensitivity to color that extends to color blindness in some taxa (*24*–*26*) and the limited use of pigments other than melanin in mammals (*2*, *4*). Genomic studies support the idea that universal color vision defects observed in crown mammals may have a Mesozoic origin (*26*, *27*). However, we ask here, why then were melanosomes from extant mammal species reported to be as diverse in geometry as colorful, mostly diurnal birds (*20*), given this early constraint in their lineage? When do diverse melanosome geometries arise in mammals?

**Fig. 1.**
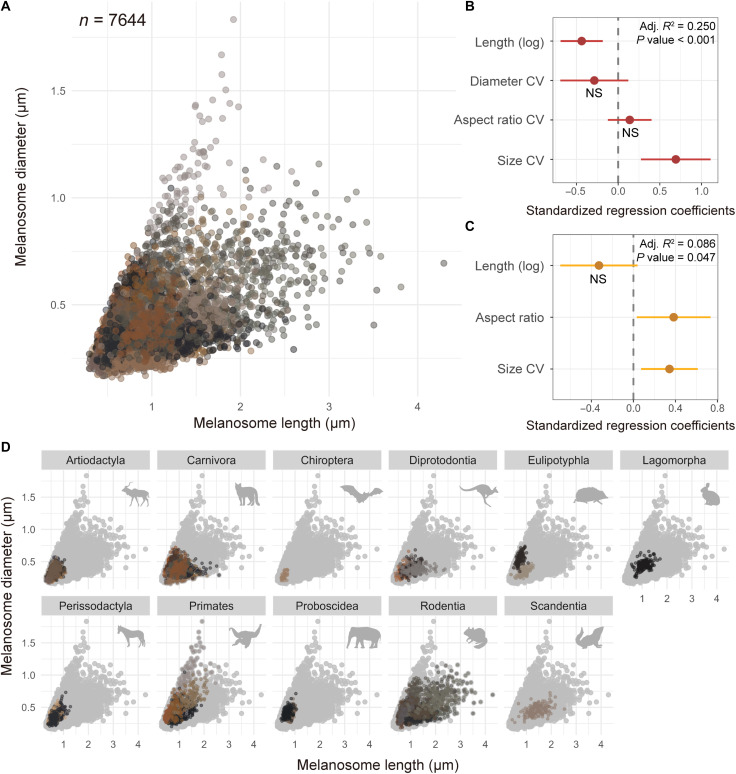
Similar melanosome geometries generate distinct colors. (**A**) Scatter plot of measured melanosomes (*n* = 7644). Results from stepwise multiple linear regression, indicating the standardized effects of melanosome variables on (**B**) *redness* and (**C**) *lightness* of the sampled pelage patches. Variation observed by mammalian order; most variation and extreme aspect ratios are observed in Rodentia (**D**). The color of the spots indicates the coloration of the sampled pelage. NS, not significant.

**Fig. 2.**
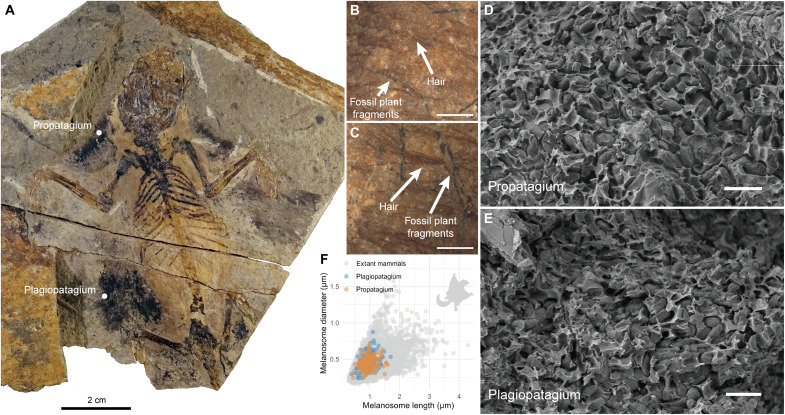
Melanosomes of the Mesozoic mammaliaform fossil species *V. diplomylos* (IMMNH-PV01699). (**A**) Main slab. The white dots indicate the positions for sampling. Scale bar, 2 cm. (**B** and **C**) Microscopic images of areas with the hair imprint in the propatagium (B) and plagiopatagium (C) of the fossil. Scale bars, 500 μm. (**D** and **E**) Scanning electron microscopy (SEM) images of melanosomes observed in the propatagium (top dot) and plagiopatagium (bottom dot) of the fossil. Scale bars, 2 μm. The more details of melanosome in these areas are presented in fig. S13. (**F**) The scatter plot of melanosomes observed in propatagium and plagiopatagium (*n*_propatagium_ = 164 and *n*_plagiopatagium_ = 145). There is no significant difference in either shape or distribution range of melanosomes between these two positions (multivariate analysis of variance: Wilks’ lambda = 0.994; *F* = 0.848; *P* = 0.429).

Here, we measured a total of 7644 individual melanosomes from 59 extant therian mammalian species across 11 orders (Fig. 1A, fig. S1, and table S1) to further investigate the relationships among melanosome geometry (figs. S2 to S6) and hair coloration in a phylogenetic context. Additionally, we sampled a Jurassic species, *Vilevolodon diplomylos* (Fig. 2) (*28*, *29*). To evaluate the potential impact of variable sample sizes for distinct species and clades, we used rarefaction analysis (figs. S7 to S10) (*30*). Digital images were used for color measurements of the sampled color patches, which is an approach standard to studies of mammal coloration, especially in cases where spectral data are unavailable or when there is a risk of color fading in museum specimens (*31*–*33*).

We quantified pelage lightness in the sampled color patch using the formula [red (*R*) + green (*G*) + blue (*B*)]/3 and assessed redness as the ratio of the R channel value to the lightness, reflecting the relative strength of red coloration (fig. S11) following published studies (*34*–*36*). Furthermore, we explored environmental variables that have been previously suggested to influence melanin deposition in mammalian pelage: Melanin is believed to enhance resistance to pathogens in warm and humid environments (*37*–*39*) and provide protection against damage from intense ultraviolet (UV) exposure (*40*, *41*). We investigated correlations with environmental variables, including temperature, precipitation, vegetation structure [represented by leaf area index (LAI)], and UV exposure levels.

## RESULTS

### Diverse melanosome geometry in modern mammals and its weak correlation with coloration

We found notable variation in melanosome geometries in specific extant therian clades, particularly within Euarchontoglires, including rodents, lagomorphs, scandentians, and primates (Fig. 1). In contrast, extant species belonging to Laurasiatheria, Atlantogenata, and marsupials displayed relatively limited variation in melanosome geometries (Fig. 1, A and D). Rodents and primates exhibited the highest variation in geometry, and some species had melanosomes that were comparable in aspect ratio to those found in iridescent birds (Fig. 1D) (*10*). Notably, squirrels had particularly long melanosomes (fig. S6), which are quite rare among extant amniotes (*10*, *20*). In birds, extremely elongate melanosomes are usually associated with photonic nanostructures (*10*), yielding iridescent structural color, but mammals with similar melanosomes geometries do not display structural coloration. The longest melanosomes were found in the rock squirrel (*Sciurotamias davidianus*, 4295 nm) (fig. S6I), while the melanosomes with the widest diameters were observed in the rhesus monkey (*Macaca mulatta*, 1833 nm) (fig. S5C).

Our data indicated a weak correlation and complex relationship between melanosome geometry and coloration in mammals (Fig. 1, B and C, and table S2), contrasting sharply with birds, where a strong correlation existed between melanosome aspect ratios and coloration (*7*, *9*). Stepwise multiple linear regression models revealed that melanosome length and coefficient of variation (CV) of melanosome size were significantly correlated with pelage redness; however, this relationship exhibited weak explanatory power [coefficient of determination (*R*^2^) = 0.250] (Fig. 1B and table S2). Similarly, the melanosome aspect ratio and size CV were significantly correlated with the pelage lightness but showed even weaker explanatory power (*R*^2^ = 0.086) (Fig. 1C and table S2).

Only three species with distinct red hair—*Ailurus fulgens*, *Pongo abelii*, and *Rhinopithecus roxellana* (figs. S3A and S5, F and G)—displayed the expected round melanosome shape reported for human red hair and associated with pheomelanin content (*42*). Other species with round melanosomes exhibited dark brown and even black coloration, such as Artiodactyla and Perissodactyla (fig. S3). Conversely, some carnivorans (Fig. 1D and figs. S3. and S4) and rodents (Fig. 1D and fig. S6) had brown or straw-colored pelage despite having elongated melanosomes. Notably, African and Asian elephants exhibited nearly identical melanosome geometries despite their pelage coloration differences (i.e., black versus brown; fig. S5, I and J).

### Hair structure and melanosome structure observed in the Jurassic species *V. diplomylos*

The fossil of *V. diplomylos* (IMMNH-PV01699, Inner Mongolia Museum of Natural History) preserved fine hair fibers from the plagiopatagium or abdomen edge and part of the propatagium (Fig. 2A). The hair fibers were distinguished from the co-occurring plant fragments by their color and structures; e.g., the fossil hair was brown, and the fossil plant fragments were black with obvious cuticles (Fig. 2, B and C, and fig. S12). Hairs were stacked and compacted as preserved, making it difficult to discern whether their types were guard hairs or underfurs.

Melanosomes were abundant, particularly in the propatagium, although it was challenging to distinguish intact individual hairs strands. This pattern of preservation seems common in Mesozoic mammalian hair (*19*). There was no significant difference observed on the melanosome geometries from the propatagium and plagiopatagium (Fig. 2F). Melanosomes in *V. diplomylos* were preserved in a disordered state, partially encapsulated in the matrix (Fig. 2, D and E, and fig. S13). Because the extant melanosomes are mostly wrapped by keratin fibers in the hair cortex (e.g., fig. S2A), this matrix could be attributed to taphonomically degraded keratinous remains; in taphonomic experiments, we found that the degradation of keratin fibers could result in the irregular arrangement and accumulation of melanosomes (figs. S12, B to C, and S13) (*43*).

The melanosomes in fossil *V. diplomylos* exhibited round and short rod shapes (mean values: length of 0.9544 μm, diameter of 0.4575 μm, aspect ratio of 2.1501, size of 0.4435 μm^2^, length CV of 0.2273, diameter CV of 0.2165, aspect ratio CV of 0.2662, and size CV of 0.3546; Fig. 2, D and E; details of melanosome geometry are in data S1). The melanosomes observed in *V. diplomylos* displayed the geometries within the range of the extant data (figs. S14 to S16) and are close to the average levels of entire extant taxa.

Our findings are consistent with data from six Mesozoic mammaliform specimens (*19*). We reached the same conclusion with different methods for melanosome measurements and different resulting values (see the “Melanosome collection and measurement” section). Specifically, we both found that the fossil melanosomes exhibit geometries within the range of the extant data, with little variation, and average values close to the average for all sampled extant taxa. In our case, values from the fossil were compared to the reconstructed ancestral state for all sampled taxa (below).

### Phylogenetic signals and ancestral states estimation of mammalian melanosome geometry

Melanosome geometries of extant mammal species show a clear phylogenetic pattern, with closely related species exhibiting similar shapes (Fig. 1). Pagel’s λ test confirmed significant phylogenetic signals for all measured geometry traits (all *P* values of <0.001; table S3), and melanosome length and aspect ratio were estimated to have evolved in patterns close to Brownian motion.

**Fig. 3.**
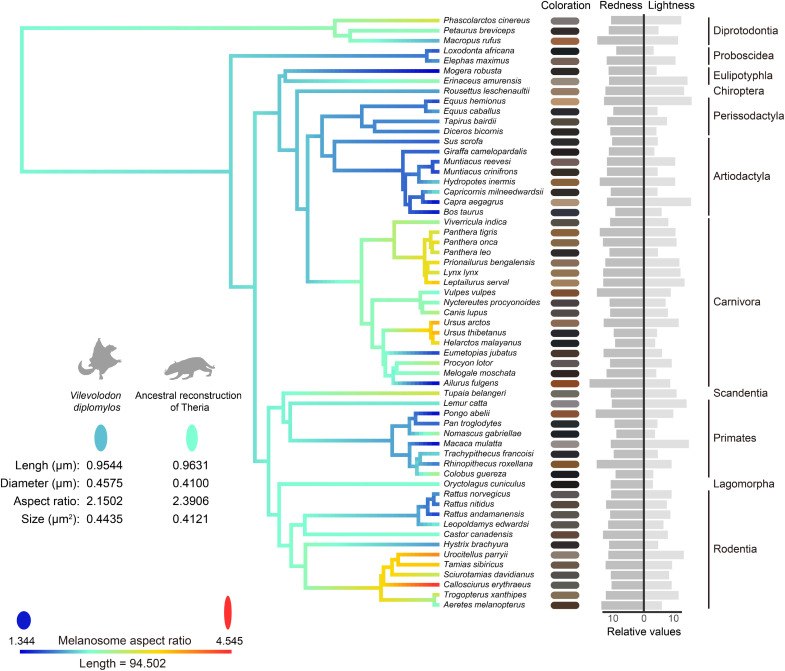
Ancestral reconstruction of mammalian melanosome geometry using Bayesian methods. The trees are color coded, with redder hues indicating higher aspect ratio values of melanosomes, while bluer tones represent lower ratios. The bands on the right side illustrate the coloration of the pelage from which the hair samples were taken, with coat coloration of each species at the tips; dark gray and light gray correspond to the relative values of coat pelage redness and lightness respectively. The melanosome geometry comparison between *V. diplomylos* and ancestral reconstruction result of Theria is located on the left.

We then reconstructed ancestral states for melanosome length, diameter, aspect ratio, and size using Bayesian Markov chain Monte Carlo (MCMC) methods. The results showed that the diversification of melanosome geometries coincided closely with the origin of major subclades of crown mammals (Fig. 3 and figs. S17 to S19). Most diversity arose comparatively recently (fig. S20). Notably, marsupials (Diprotodontia) exhibited limited geometric variation, with trait values resembling ancestral states for deep nodes (Figs. 1 and 3). Among placentals, only elephants (Atlantogenata) showed a similarly low diversity, while other taxa displayed clear disparities in geometry from estimated ancestral states. The observed melanosome geometries of *V. diplomylos* closely resemble the deep node (Theria) states of our ancestral state reconstructions (Fig. 3), although *V. diplomylos* diverged from other stem mammals well before the Theria ancestor (*28*, *29*).

### Assessing the relationship between environmental variables and melanosome geometry in a phylogenetic framework

**Fig. 4.**
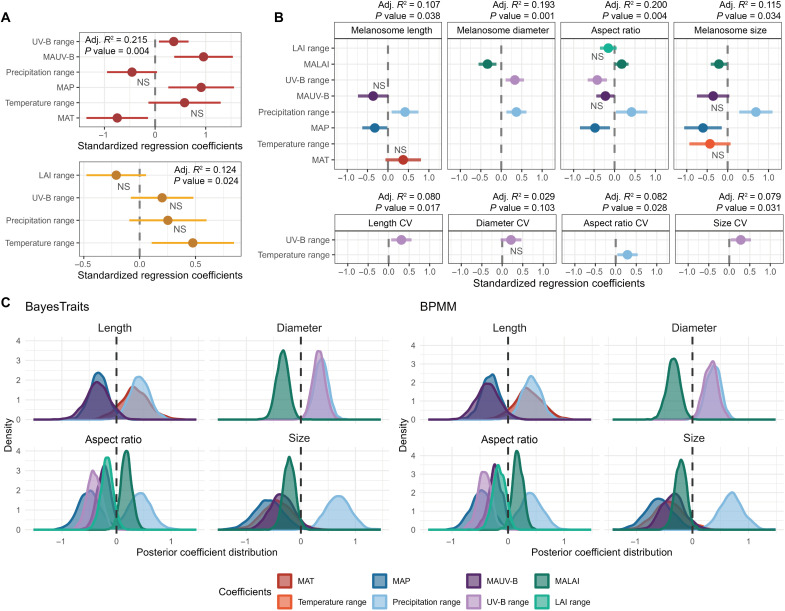
The relationships between environmental factors and mammalian hair color and melanosome geometries. (**A**) Coefficients of effects explaining the coloration parameters using stepwise multiple linear regression, indicating the standardized effects of environmental variables on redness (red) and lightness (yellow). (**B**) Standardized effects of environmental factors on the melanosome geometries and corresponding CVs using the stepwise PGLS model based on the maximum clade credibility tree. NS, not significant. (**C**) Phylogenetic Bayesian posterior distributions from the BayesTraits models and Bayesian phylogenetic mixed model models estimate the effects of environmental variables on the melanosome geometries using 1000 trees block. Abbreviations for environmental variables: *MAT*, mean of annual temperature; *MAP*, mean of annual precipitation; *MAUV-B*, mean of annual ultraviolet-B radiation; *MALAI*, mean of annual leaf area index. The error bars indicate 95% confidence intervals.

To evaluate whether select environmental variables affect the melanosome geometries in crown mammals, we built phylogenetic generalized linear models for melanosome geometry [phylogenetic generalized linear models (PGLS, table S4)]. We used annual average values as proxies for typical environment and selected the amplitude of annual variation as a proxy for seasonal influence (*44*). For comparison, we also built stepwise multivariate linear regression models for hair color to evaluate the effects of environmental factors on coloration. Our results revealed different recovered relationships between environmental variables and melanosome geometry and between these variables and hair coloration (Fig. 4 and tables S4 to S7).

Annual precipitation, including mean annual precipitation (MAP) and its annual range, was recovered as significantly correlated with all four melanosome geometry measurements examined (*P* values and *R*^2^ values are given in Fig. 4B and table S4). Specifically, increased annual precipitation range was positively correlated with more elongated and larger melanosomes, whereas smaller and more spherical melanosomes were observed in species consistently inhabiting humid environments. Both maximum likelihood and Bayesian modeling methods (BayesTraits and Bayesian phylogenetic mixed model) confirmed the PGLS model results (Fig. 4C and tables S5 and S6).

The annual range in UV-B radiation, rather than mean annual UV-B (MAUV-B), was another significant environmental factor affecting melanosome geometry (Fig. 4B and table S4). A higher UV-B range was positively associated with larger melanosome diameter and lower aspect ratio. Additionally, increased UV-B range contributed to greater heterogeneity in melanosome shape, resulting in higher CVs (Fig. 4B). Because LAI negatively regulates UV exposure, higher mean annual LAI (MALAI) correlated with smaller, elongated melanosomes (Fig. 4B), showing an inverse effect to that of UV-B range. However, the range value of LAI did not exhibit any significant influence (all *P* values of >0.05, tables S4 and S7).

The most significant difference in the influence of environmental factors on melanosome geometry versus coloration was with respect to temperature. Both redness and lightness were significantly correlated with temperature (Fig. 4A), aligning with many observations in the literature (*34*, *36*, *45*). However, melanosome shape was not correlated with environmental temperature (Fig. 4B). MAP showed a positive correlation with redness (Estimate_redness-MAP_ = 0.909, *P* = 0.008), consistent with results obtained from South American rodents (*34*); in contrast, lightness did not exhibit any significant relationship with precipitation related factors (Fig. 4A and table S7). MAUV-B positively correlated with redness (Estimate_redness-MAUV-B_ = 0.956, *P* = 0.017; Fig. 4A and table S7).

## DISCUSSION

The diversification of therian mammalian melanosome geometries primarily occurs within major subclades of crown placental mammals (Fig. 1 and S19), which may correlate with the early Cenozoic rapid radiation of these clades (*19*, *46*). The similarity in melanosome geometry between *V. diplomylos* and reconstructed ancestral states further supports the conclusion that Mesozoic mammals may have a more limited range for melanosome geometry (Fig. 3 and figs. S16 to S18) (*19*). Given the phylogenetic distance between the *V. diplomylos* and estimates for the ancestor of Theria (*28*, *29*), it appears that these restricted melanosome geometries could have persisted for at least tens of millions of years. Thus, Mesozoic mammals likely exhibited limited color variation and expressed predominantly dark pelage (*19*). This pattern contrasts sharply with that observed in Pennaraptoran dinosaurs and pterosaurs, which displayed high diversity and geometric specialization of melanosomes during their initial appearance in the Jurassic (*8*, *9*, *11*, *17*, *18*). In birds and feathered dinosaurs, melanosomes with extreme lengths and diameters are often associated with structural coloration, which is driven by the requirements for organized layering (*10*, *17*). These findings suggest that dinosaurs, birds, and even pterosaurs in the Mesozoic had a diverse range of color expression (*8*, *9*, *11*, *17*, *18*) that likely corresponded with their acute color vision.

Relaxed selection for color-melanosome geometry relationships may also explain the observed pattern (Figs. 1 and 4) in mammals. We find the melanosomes contained in extant brown and black mammalian hair are often similar in geometry, and geometry is recovered as weakly related to color (Fig. 1 and fig. S1). Mammals do not display the vivid structural colors associated with melanosomes of extreme lengths or diameters (Fig. 1). The mammalian species (such as squirrels and some primate species, figs. S5 and S6), which have melanosome with extreme aspect ratio or size, are not beyond the common coloration, indicating a more relaxed restriction on melanosome shape in Therians. Accordingly, most mammals are dichromatic (*26*, *27*), with some nocturnal species having completely lost color vision (*47*) and differences in color sensitivity between mammals and birds are thought to have originated during the Mesozoic (*48*). The nocturnal bottleneck hypothesis posits that ancestral mammals and mammaliaforms lived in low-light conditions, resulting in diminished color vision (*49*). Recent evidence of uniform coloration from an array of Mesozoic mammals (*19*) further supports a role for the nocturnal bottleneck in limiting color in mammals. Extant mammals generally lack the green, blue, and purple hues with species that regained trichromatic vision developed structural coloration in their skin, instead of hair (*50*).

Early studies observed that feathered dinosaurs, birds, and mammals exhibit substantially larger variation in melanosome shape compared to other amniotes (*20*), a feature attributed to pleiotropic effects of the melanocortin system and parallel shifts in *MC1R* in both clades in some studies (*51*, *52*). They proposed that higher metabolic rates may correlate with greater melanosome diversity, which a correlation supported in limited subsequent studies (*20*, *30*, *53*). Our results, confirming melanosome shape variability on the scale only otherwise known in birds, support unique similarity between these two clades. Our recovery of different relationships between color and shape in mammals from those reported in birds may also be consistent with these previous studies reporting distinct relationships between metabolic rate and melanosome shape in these two clades (*53*). More generally, however, our findings underscore the need for further research into controls on melanin content and geometry in mammalian melanosomes. Studies of *Pmel*-inactivated mice have experimentally shown that melanosome shape can vary independently of associated pheomelanin content (*54*). Additionally, studies of *Agouti*, which influences the density of melanosomes in mammalian hair (*55*), could provide further insights into this complex relationship.

Our PGLS models found that hair coloration and melanosome geometry are influenced by distinct environmental factors likely through distinct mechanisms. While melanosome geometry shows little relationship to temperature, it demonstrates significant responsiveness to other variables such as precipitation and UV radiation (Fig. 4B). However, these variables, like hair color, explained only limited amounts of the observed variation (Figs. 1 and 4). However, some taxa also reflected a more strong relevant between coloration and both environmental and geographic variables (*35*, *45*), which indicate a complex pattern in Mammalia. Given that the overall results of PGLS model are significant, for some taxa, the influence of ecological factors will be higher than the average level. However, for some other taxa, the situation may be the opposite.

Insight from melanosome geometries in fossils, with increased data and future insight into other variables affecting this relationship, may allow comparisons with paleoenvironmental inferences from independent data. For example, the geometry of melanosomes in the Jurassic fossil *V. diplomylos* mostly closely resembles that of modern species such as *Melogale moschata*, *Rattus nitidus*, and *Petarurus breviceps* (fig. S1; for details of melanosome geometry, see data S2). While melanosome shape is strongly influenced by phylogenetic relationships in extant mammals, the habitats of these three modern species indicate that they occur in conditions characterized by moderate precipitation and seasonal changes, accompanied by relatively high UV radiation (data S2).

Paleoenvironmental data indicate that *V. diplomylos* inhabited a late Middle Jurassic forest (*56*, *57*), located at mid-latitudes (~30°N to 40°N) (*58*). Ecological reconstruction based on fossil insect communities indicated that this forest experienced a warm and humid climate, with moderate annual rainfall (MAP at 1500 to 2000 mm) and relatively higher UV radiation levels (region altitude was ~1000 to 1500 m) (*59*). Distinct tree rings observed in the associated wood fossil suggested seasonal variations (*60*, *61*). In this case, the signal in these two types of data is largely consistent.

In conclusion, we report obvious differences between the relationship between color and geometry in mammals from those explored in birds. We also explored evidence for links between melanosome geometry and environmental variables that have not yet been investigated in birds. Future studies should aim to further enhance our understanding of the complex relationships among melanin chemistry, color, environment, activity pattern, and physiology in mammaliaform taxa.

## MATERIALS AND METHODS

### Melanosome collection and measurement

#### Samples from extant species

We collected hair samples from a total of 59 modern mammalian species spanning 11 orders, including three marsupial species, for melanosome measurement (table S1 and figs. S2 to S6). The specimen hair samples were collected from mammalian coats directly, while the living mammalian hair samples were taken from shedding hair naturally. All the collected hairs were washed with ethanol and dried before measurement. We used clean razor blades to scrape the surface of the hair gently until the cuticle was destroyed, causing the keratin fibers to tear open and expose the melanosomes. The collection and experiments were reviewed and approved by the Nanjing University Committee on Science and Technology Ethics (reference code: OAP20240718001).

Unlike the ultrathin sectioning method described by Li *et al.* (*19*), this method preserves the three-dimensional geometry of melanosomes and minimizes inaccuracies in measurement data caused by angular tilt, especially for larger melanosomes (e.g., those from Sciuridae). After preparation, the samples were observed and documented using field emission scanning electron microscopy (SEM; ZEISS GeminiSEM 360). The accelerating voltage was set to 1.5 kV to prevent potential excessive discharge.

#### Fossil specimen and provenance

The fossil specimen of *V. diplomylos* (IMMNH-PV01699) is a skeleton with partial skull, mandible, and most of the postcranial skeleton preserved. This specimen was collected from Nanshimen site of the Tiaojishan Formation in Qinglong County, Hebei Province, China, by Junyou Wang in 2017. This specimen was estimated to be 161 to 160 million years old on the basis of stratigraphical correlation with the index fossil *Qaidamestheria* (*29*). This specimen is accessioned at Inner Mongolia Museum of Natural History, which is accessible to researchers.

For fossil samples, the melanosomes were already exposed, so we did not perform any specific pretreatment before measurement. To test the potential effects of melanosome diversity across different organs on geometric measurements (*62*), we selected two distinct locations of *V. diplomylos* (the edge of the propatagium and the plagiopatagium, Fig. 2A) and compared the shapes of melanosomes at these two positions. Detailed information on both modern and fossil melanosome geometries is provided in the supplementary files (data S1 and S3).

For all species, we measured melanosome lengths and diameters using SEM images analyzed with ImageJ version 1.54f (*63*). We then calculated both the aspect ratio (*length*/*diameter*) and longitudinal section size (*length* by *diameter*, as a brief representation of volume) for each individual melanosome. These values were then used to determine the mean values and CV. To assess whether our data were influenced by sample size bias, especially regarding the CVs, we evaluated the impact of sample size using the rarefaction curve (figs. S7 to S10). We ensured that the sample size for each species exceeded the requirements for the parameters to reach an asymptote (*30*).

### Color data

Considering the potential for color distortion under microscopy and the natural fading of colors for the specimens deposited in museums, we chose to collect digital images of each mammalian species to analyze their coat colors. Digital images are widely used in animal coloration studies (*31*–*33*) and correlate reliably with data from standardized photography and spectrophotometry. Therefore, digital images are the logical choice, particularly in cases where spectral data are unavailable.

These images were compiled from the open-access sources, including iNaturalist, Nature Picture Library, American Society of Mammalogists, Animal Diversity Web, and Google Images. At least five images were selected to enhance the reliability of the results for each species (except in the case of *Aeretes melanopterus* for which only three reliable images were found). We excluded the images taken against dim or bright backgrounds and avoided shadowed areas for color collection, especially for the black coated mammals, to reduce the disturbance of environmental ambient light. For species that exhibit bicolor or multicolor patterns, we focused only on the color of the regions from which the hair samples were collected. We extracted raw RGB (red, green, and blue) values from ImageJ and calculated mean channel values (fig. S11).

Mammalian coat color is primarily determined by two pigments: eumelanin, which produces shades of dark brown to black, and pheomelanin, which produces shades of yellow to red (*4*, *21*). Therefore, we transformed the raw RGB values to two parameters, lightness and redness, to quantify the relative levels of eumelanin and pheomelanin in mammalian hair respectively. We followed the methods described by previous studies (*32*, *34*–*36*) and quantified the *lightness* as (*R* + *G* + *B*)/3 and *redness* as the ratio of the R channel value to lightness to reflect the relative strength of redness (fig. S11). In our dataset, species with redness exceeding 1.4 display a visually distinct red hue, while those with redness close to or lower than 1.0 appear predominantly black or gray.

### Environmental and ecological data

We collected temperature and precipitation data to construct regressions for mammalian coloration and melanosome geometry. To acquire reliable species-level geographic maps for the extracted environmental factors, we obtained mammalian spatial data in the form of Environmental Systems Research Institute shapefiles (polygons) from the International Union for Conservation of Nature (IUCN). These shapefiles represent the comprehensive expert knowledge database on species ecology and distribution. To facilitate subsequent analyses, we converted the polygon range maps into an equal-area grid at 0.5° by 0.5° resolution (Behrmann projection, about 50 km at the equator). These polygons were loaded into R and manipulated therein using the R packages terra and sf (*64*).

For the domesticated species not in the IUCN Red List, *Equus caballus* and *Sus scrofa*, we used occurrence data from the Global Biodiversity Information Facility (GBIF) as a substitute for polygon maps. The loading and manipulation of GBIF data were used using the R package rgbif (*65*). Environmental data were downloaded as raster files from WorldClim (version 2.1) database (https://worldclim.org/) at a 2.5–arc min resolution (*66*). We selected six bioclimatic variables to describe the habitats: BIO1 [mean annual temperature (MAT)], BIO5 (maximum temperature of the warmest month), BIO6 (minimum temperature of the coldest month), BIO12 (MAP), BIO13 (precipitation of the wettest month), and BIO14 (precipitation of the driest month). Because of collinearity between extreme and mean values, we computed the annual range for these two groups of factors by subtracting the maximum value from the minimum value. In brief, we introduced two extra parameters: change range of annual temperature (BIO5 and BIO6) and change range of annual precipitation (BIO13 and BIO14) to replace the extremes.

In addition to temperature and precipitation, we included monthly UV-B radiation levels and LAI data from a global high-resolution database (*67*, *68*). UV radiation is recognized as a strong predictor of the melanin-related coloration in animals (*41*), because photoprotection is considered to be one of the most important functions of melanin in vertebrates (*3*, *69*). Therefore, radiation intensity may potentially influence the melanosome geometry. LAI, a key parameter for forest primary production estimation, is linked to the immune function of melanin (*32*, *37*).

Additionally, LAI helps to estimate the degree of shade caused by forests and shrublands, influencing the amount of solar radiation reaching the ground (*70*). Simultaneously, LAI indicates habitat openness levels (*71*, *72*); thus, we set it as a combined environmental influence factor. Consistent with the treatment of temperature and precipitation, we obtained the MAUV-B radiations and MALAI values, along with differences between monthly maximum and minimum values as change ranges for analysis. All values were computed as averages of each 0.5° grid cell, with environmental factors corresponding to individual species represented by averages from all the grid cells covering their habitats. For species with only occurrence data, we documented environmental factor values at each occurrence point and calculated their average.

### Analysis of color

To evaluate the relationship between melanosome geometry, environmental factors, and coloration, we established a series of stepwise multivariate linear regression models using the function step in the R package stats. This function determines the best-fitting model based on Akaike information criterion (AIC) scores. For geometry, eight parameters (*length*, *diameter*, *aspect ratio*, *size*, and their CVs) were set as the explanatory variables and the redness and lightness as the response variables, respectively. Because the contribution of parameter size can be jointly explained by variation in length and diameter, it was excluded in stepwise regressions.

After we obtained the best model of redness and lightness, we tested the multicollinearity of models using the variance inflation factor (VIF) via the vif function in the R package car. The length CV (in redness model) and aspect ratio (in lightness model) were observed excessively high VIF values (VIF_length CV_ = 27.90 and VIF_aspect ratio_ = 39.03); therefore, we removed these two parameters separately. After removing high VIF variates, we repeated VIF test, and the final models achieved VIF values all below 10 with the similar *R*^2^. To ensure whether the relationships between each explanatory variable and the response variable in the final models were consistent with the statistical test, we checked the partial residual plots for detection using functions in the R package car (fig. S21).

Similarly, for environmental factors, we selected another eight variables (*MAT*, *MAP*, *MAUV-B*, *MALAI*, and their ranges) and performed stepwise regressions to examine their relationships with coloration. Length, diameter, and size were log transformed, and all variables were scaled. The VIF values for the models were all below 10. Q-Q plots and partial residual plots were also checked (fig. S22).

### Phylogenetic comparative analysis

We used the latest large-scale mammalian phylogenies for analysis from the VertLife project (*73*), which covered all the taxa of our samples. We obtained 1000 trees from the posterior distribution randomly using the online tool (https://vertlife.org/phylosubsets/) and pruned them down to the 59 mammals that we studied. We used the TreeAnnotator tool from BEAST version 2.7.5 (*74*) to estimate the maximum clade credibility tree of these 1000 trees. In addition, to evaluate the uncertainty of phylogenetics, we also sampled a 100 trees subset randomly from the 1000 trees.

First, we tested the phylogenetic signals of redness, lightness, and all melanosome geometry factors by applying Pagel’s λ (*75*) using the function phylosig in the R package phytools (*76*), which is used to measure the phylogenetic independent levels of traits. When values approach 0, it suggests that the trait evolved independently of phylogeny, whereas values close to 1 indicate evolution followed the Brownian motion. Pagel’s λ indicated that color factors (redness and lightness) and CVs (CVs of diameter, aspect ratio, and size) were not phylogenetically conserved (table S2). Then, we performed a follow-up phylogenetic comparative analysis for four main geometrical factors with significant phylogenetic signals: length, diameter, aspect ratio, and melanosome size, using linear models (for statistical hypothesis testing, we excluded the high–leverage point *P. abelii* from the size CV model). We reconstructed the ancestral states of these four factors by Bayesian MCMC methods using the function anc.Bayes in the R package phytools. The traits and tree visualizations were plotted by the R packages ggtree (*77*) and ape (*78*).

To evaluate how the ecological factors affect the shape of melanosomes in phylogenetic framework, we built the stepwise PGLS using the function phylostep in the R package phylolm (*79*), which estimate the best-fitting model by the AIC rank when the penalty parameter *k* = 2. Given the relatively small sample size in our study, we only estimated the best-fitting models by the Brownian model with scaling factors (lambda) to avoid the type I error rates caused by Ornstein-Uhlenbeck models with limited tips (*80*). Because the VIF test is not included in the PGLS model (*81*), we evaluated the multicollinearity of the best-fitting PGLS models on the standard linear models with the same formulas and ensured the VIF values of all of the explanatory variables were acceptable (VIF < 10). To verify the uncertainty of the phylogeny, we repeated all of the models of the four variables related to melanosome geometry on the 100 trees subset independently and calculated the model averages and 95% confidence intervals of all variates. The repeated operations of the models were programed by the R packages ape, geiger (*82*), and nlme (*83*).

We also fitted all the regression models using the “Continuous” program in BayesTraits version 4.1.1 (*84*). This program allowed us to generate the posterior distributions of PGLS, and we could estimate the coefficients and scaling parameters lambda of regressions by the MCMC method. To account for the uncertainty of phylogeny, we used a 1000 trees block to generate the posterior distributions. This analysis was run for 2 million iterations, and the first 200,000 iterations (10%) were burned-in. Because of the collinearity in neighboring iterations, samples were taken every 500 iterations. The results of BayesTraits were loaded on and analyzed by the R packages bayestraitr (*85*) and coda (*86*). We obtained the mean and Bayesian 95% high probability density (HPD) intervals of all variates and used the probability parameter (*P*_MCMC_) to assess their significance.

To examine the results of the linear models and BayesTraits, we used the MCMC general linear mixed models (MCMCglmm) and checked it again using the R package MCMCglmm (*87*). This method accounts for the phylogenetic nonindependence by setting the phylogenetic relationship as the random effect for regression; thus, we could evaluate both the phylogenetic effects and other potential effects (*88*). We fitted the models with the uninformative inverse-Wishart priors (*V* = 2 for random effects and ν = 0.002 for residual covariance) and run MCMC chains for 150,000 iterations. We discarded the initial 50,000 iterations as burn-ins and sampled every 500 iterations thereafter. To test the convergence and noncollinearity, we ran the Gelman and Rubin’s diagnostic (*89*) using gelman.diag in coda and inspected the trace using mcmc_plot in sirt. Similarly, for the uncertainty of phylogeny, we fitted each model to each of the 100 trees, and the coefficient averages and the 95% HPD intervals of all models were estimated from the entire jointed pseudo-posterior distribution.
