# Supplementary material for: High melanosome diversity exhibits weak correlation with color and environmental variables in the early evolution of therian mammals
Source: Sci Adv. 2025 Oct 24;11(43):eadw8707. doi: 10.1126/sciadv.adw8707 (PMC12551707; doi:10.1126/sciadv.adw8707)
Supplement: Supplementary file 1 — Figs. S1 to S22 Tables S1 to S7 Legends for data S1 to S3 [file sciadv.adw8707_sm.pdf]

Science Advances
AAAS

Supplementary Materials for

High melanosome diversity exhibits weak correlation with color and environmental variables in the early evolution of therian mammals

Xin Li *et al.*

Corresponding author: Yanhong Pan, panyanhong@nju.edu.cn; Shundong Bi, sbi@iup.edu

Sci. Adv. **11**, eadw8707 (2025)
DOI: 10.1126/sciadv.adw8707

The PDF file includes:

Figs. S1 to S22
Tables S1 to S7
Legends for data S1 to S3

Other Supplementary Material for this manuscript includes the following:

Data S1 to S3

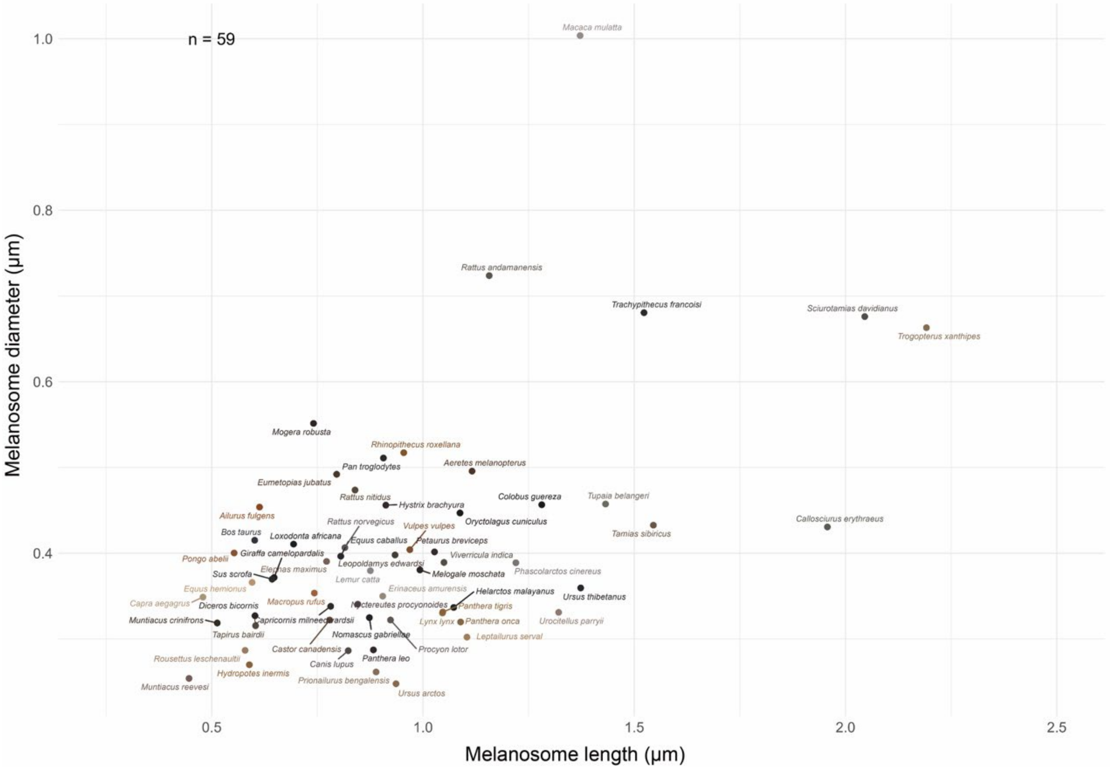

Fig. S1.

The scatter plot of the average melanosome geometry of each species. The color indicates the average color of pelage where the hair sample was taken from for each species.

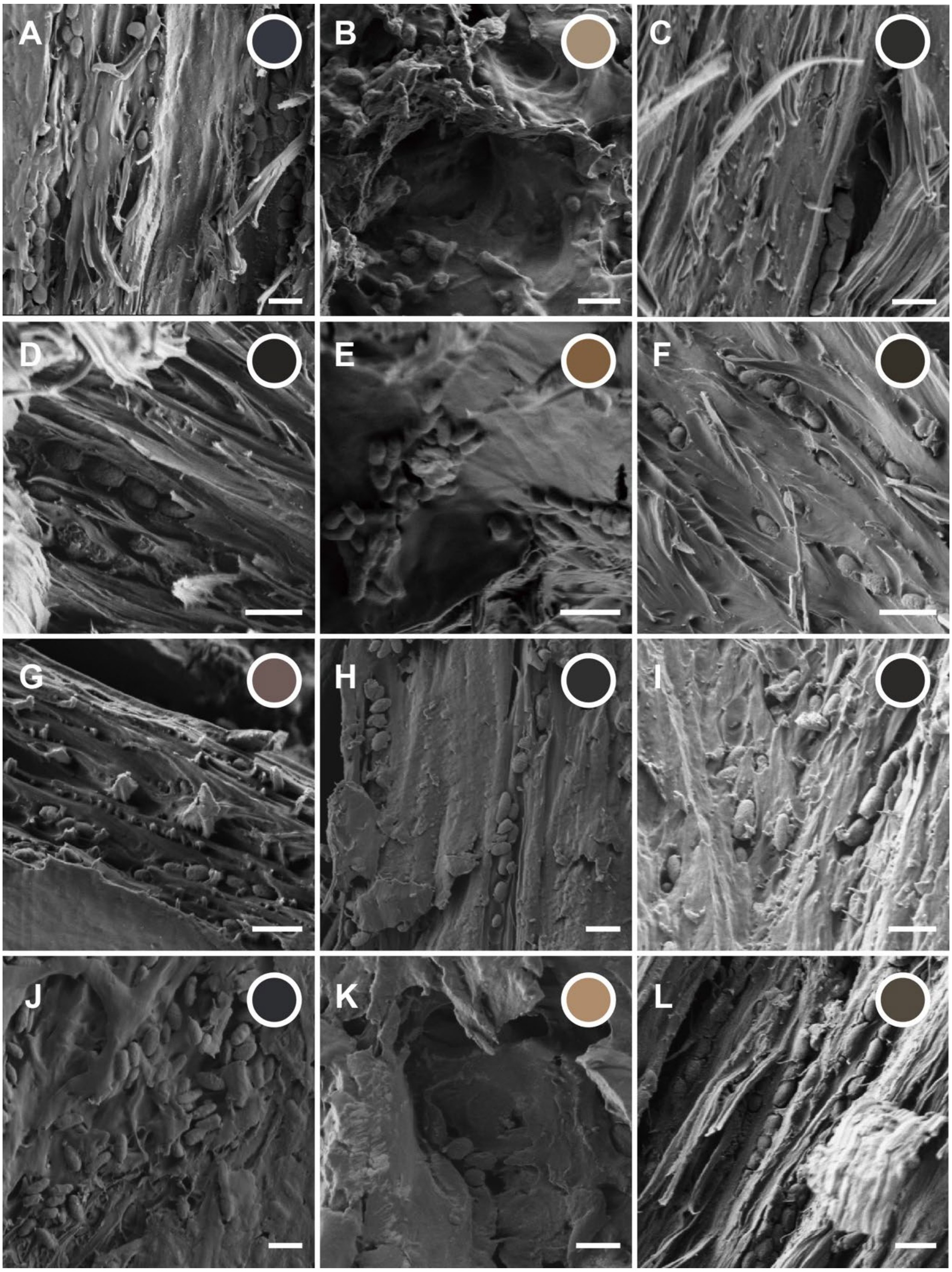

Fig. S2.

SEM pictures of Artiodactyla (A-H) and Perissodactyla (I-L) Melanosomes. (A) *Bos taurus*; (B) *Capra aegagrus*; (C) *Capricornis milneedwardsii*; (D) *Giraffa camelopardalis*; (E) *Hydropotes inermis*; (F) *Muntiacus crinifrons*; (G) *Muntiacus reevesi*; (H) *Sus scrofa*; (I) *Diceros bicornis*; (J) *Equus caballus*; (K) *Equus hemionus*; (L) *Tapirus bairdii*. Scale bar: 1 μm. The color of circle in the upper right corner indicates the pelage average color where hair sample from.

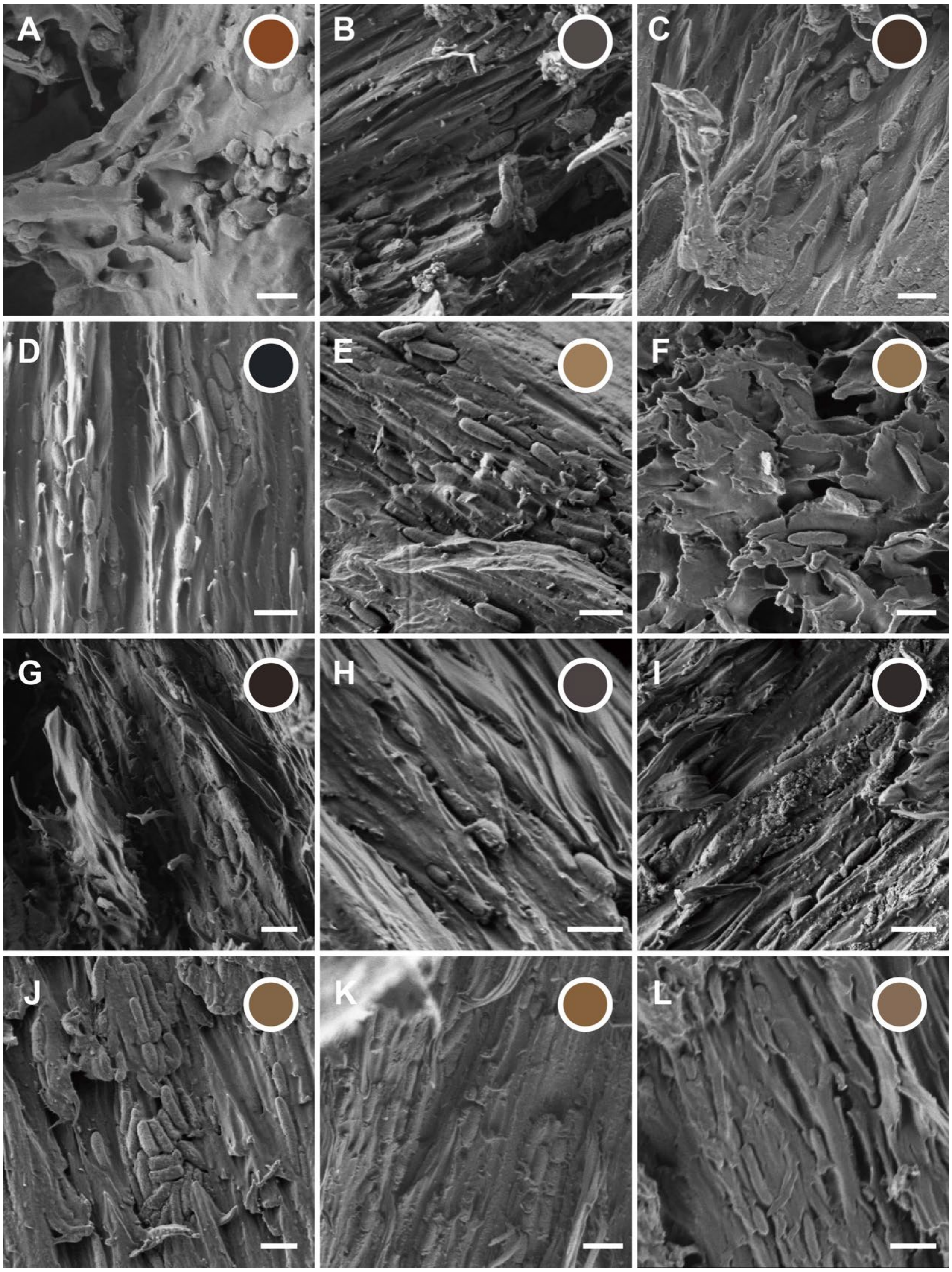

Fig. S3.
SEM pictures of Carnivora Melanosomes. (A) *Ailurus fulgens*; (B) *Canis lupus*; (C) *Eumetopias jubatus*; (D) *Helarctos malayanus*; (E) *Leptailurus serval*; (F) *Lynx lynx*; (G) *Melogale moschata*; (H) *Nyctereutes procyonoides*; (I) *Panthera leo*; (J) *Panthera onca*; (K) *Panthera tigris*; (L) *Prionailurus bengalensis*. Scale bar: 1 µm. The color of circle in the upper right corner indicates the pelage average color where the hair sample was taken from.

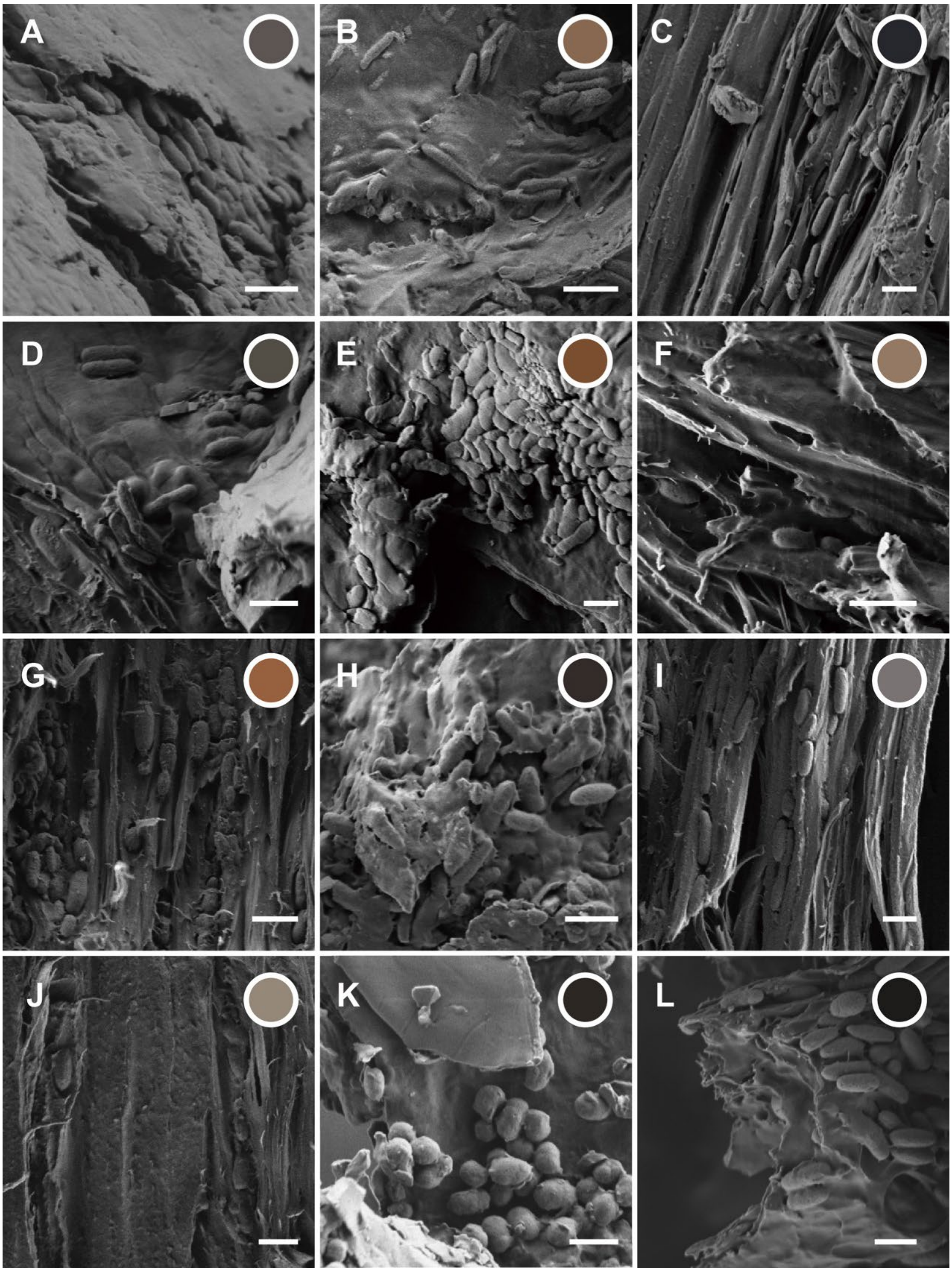

Fig. S4.

SEM pictures of Carnivora (A-E), Chiroptera (F), Diprotodontia (G-I), Eulipotyphla (J and K) and Lagomorpha (L) Melanosomes. (A) *Procyon lotor*; (B) *Ursus arctos*; (C) *Ursus thibetanus*; (D). *Viverricula indica*; (E) *Vulpes vulpes*; (F) *Rousettus leschenaultia*; (G) *Macropus rufus*; (H) *Petaurus breviceps*; (I) *Phascolarctos cinereus*; (J) *Erinaceus amurensis*; (K) *Mogera robusta*; (L) *Oryctolagus cuniculus.* Scale bar: 1 μm. The color of circle in the upper right corner indicates the pelage average color where the hair sample was taken from.

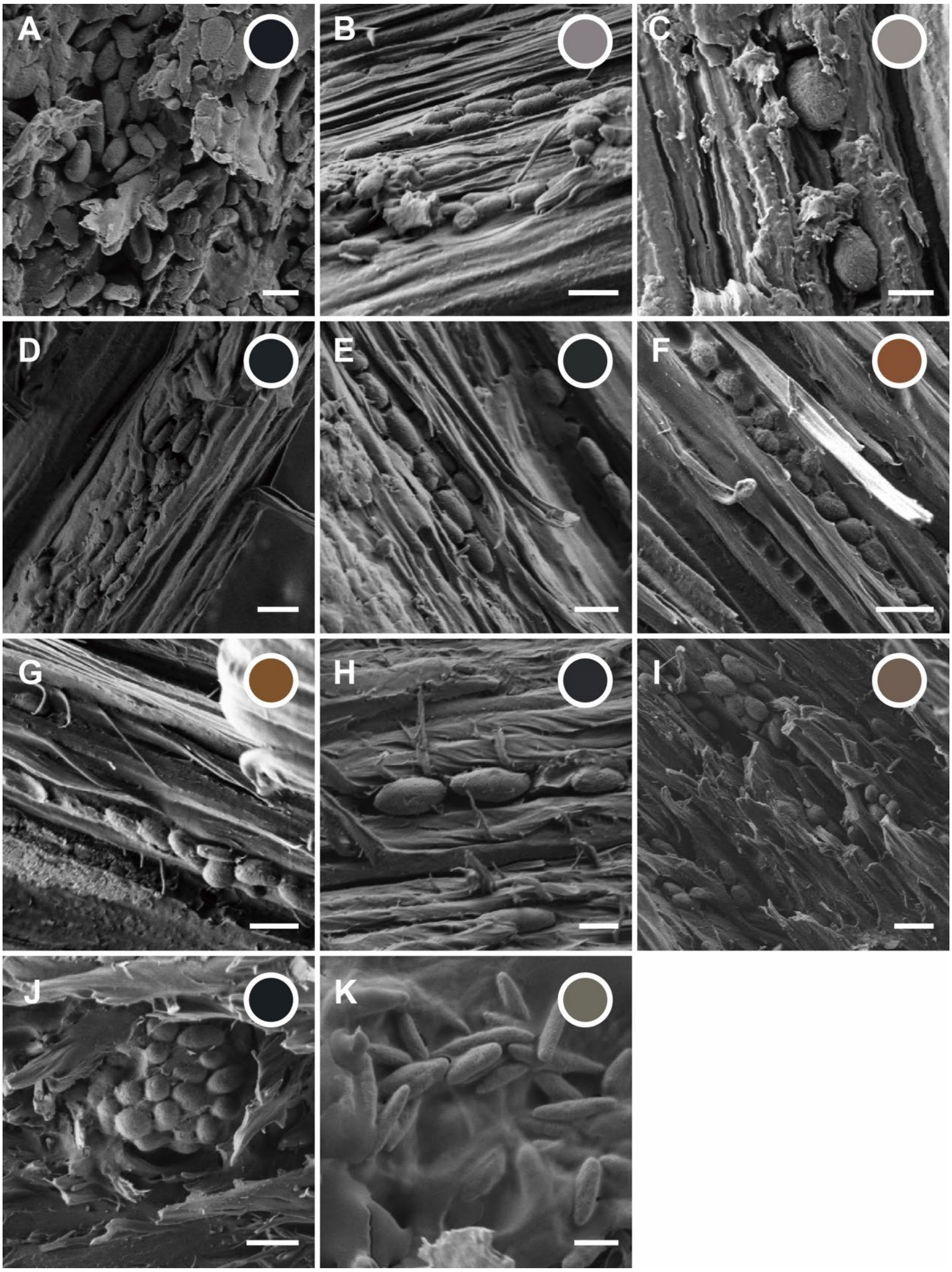

Fig. S5.
SEM pictures of Primates (A-H), Proboscidea (I and J) and Scandentia (K) Melanosomes. (A) *Colobus guereza*; (B) *Lemur catta*; (C) *Macaca mulatta*; (D) *Nomascus gabriellae*; (E) *Pan troglodytes*; (F) *Pongo abelii*; (G) *Rhinopithecus roxellana*; (H) *Trachypithecus francoisi*; (I) *Elephas maximus*; (J) *Loxodonta africana*; (K) *Tupaia belangeri*. Scale bar: 1 µm. The color of circle in the upper right corner indicates the pelage average color where the hair sample taken from.

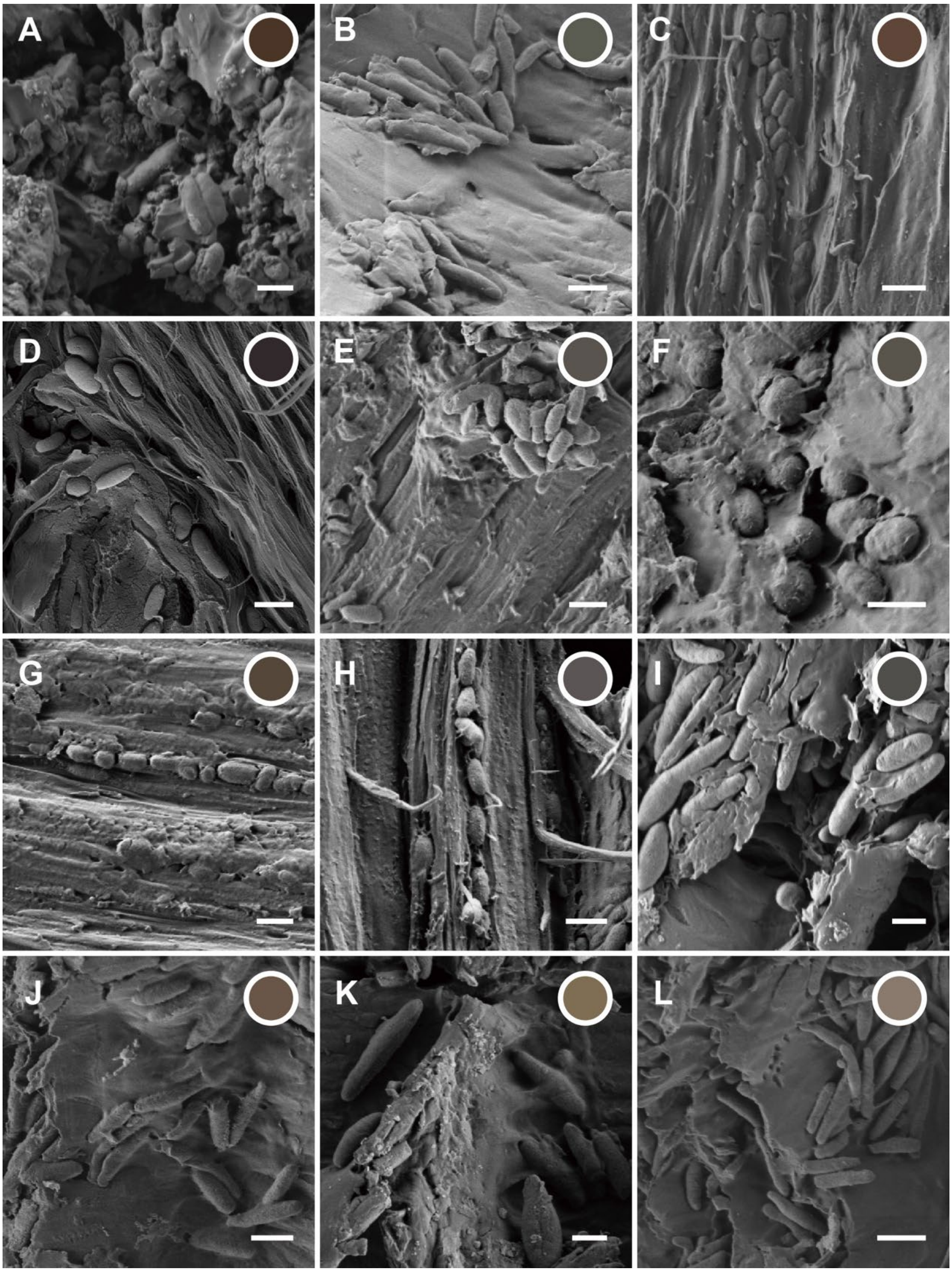

Fig. S6.

SEM pictures of Rodents Melanosomes. (A) *Aeretes melanopterus*; (B) *Callosciurus erythraeus*; (C) *Castor canadensis*; (D) *Hystrix brachyura*; (E) *Leopoldamys edwardsi*; (F) *Rattus andamanensis*; (G) *Rattus nitidus*; (H) *Rattus norvegicus*; (I) *Sciurotamias davidianus*; (J) *Tamias sibiricus*; (K) *Trogopterus xanthipes*; (L) *Urocitellus parryii*. Scale bar: 1 μm. The color of circle in the upper right corner indicates the pelage average color where the hair sample was taken from.

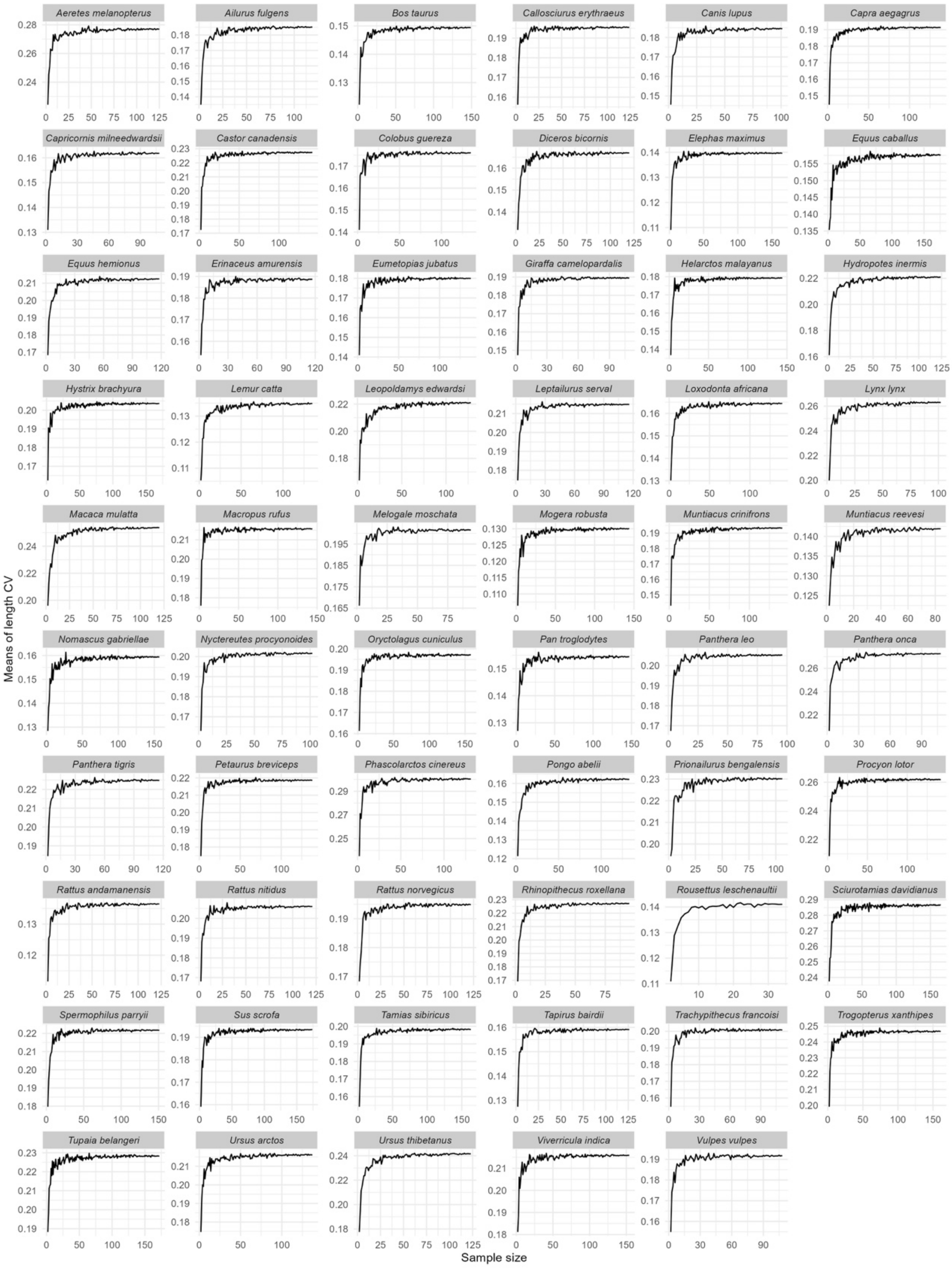

Fig. S7.

The rarefaction curves of sample size to the melanosome length CVs.

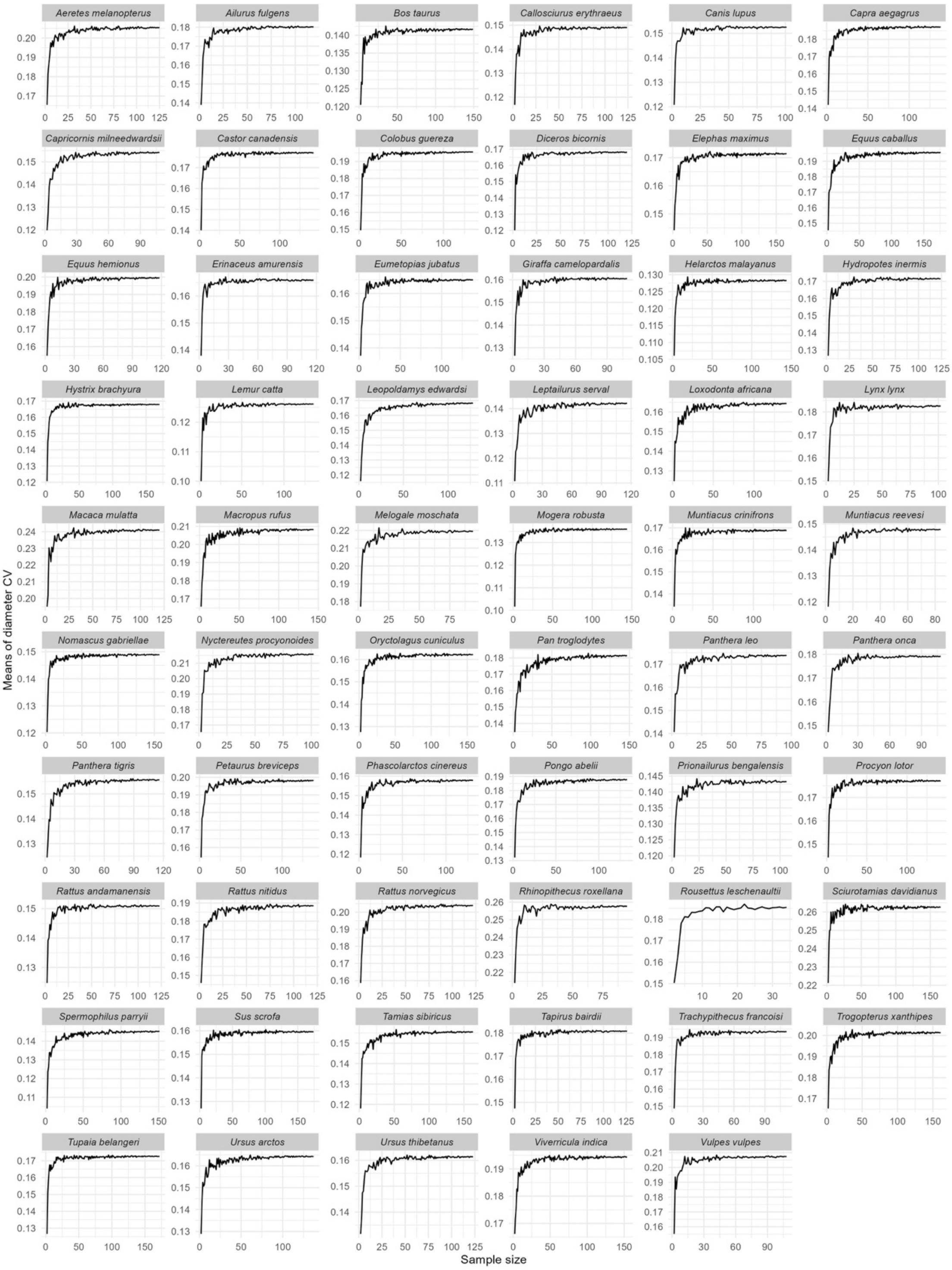

Fig. S8.

The rarefaction curves of sample size to the melanosome diameter CVs.

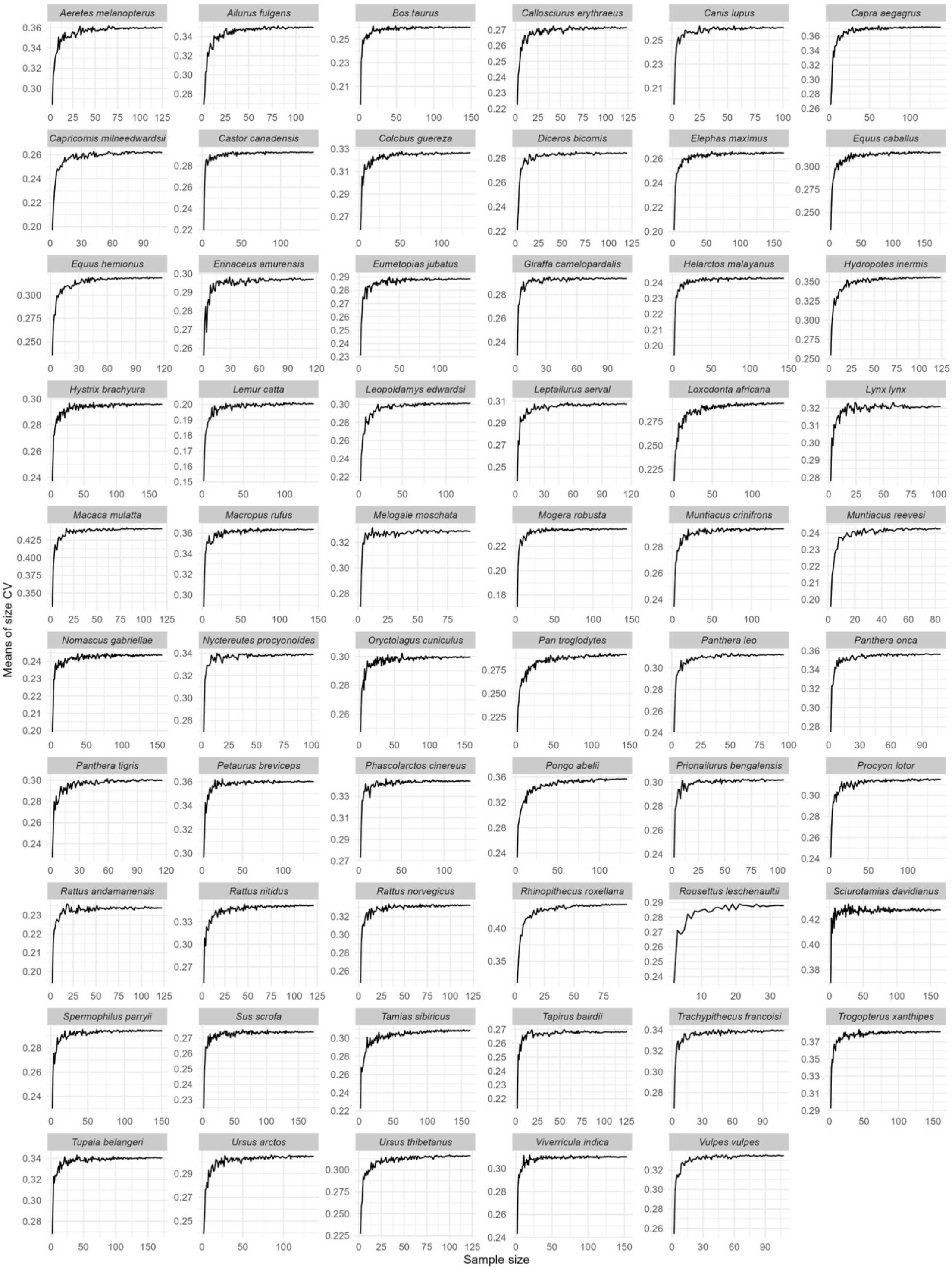

Fig. S9.

The rarefaction curves of sample size to the melanosome size CVs.

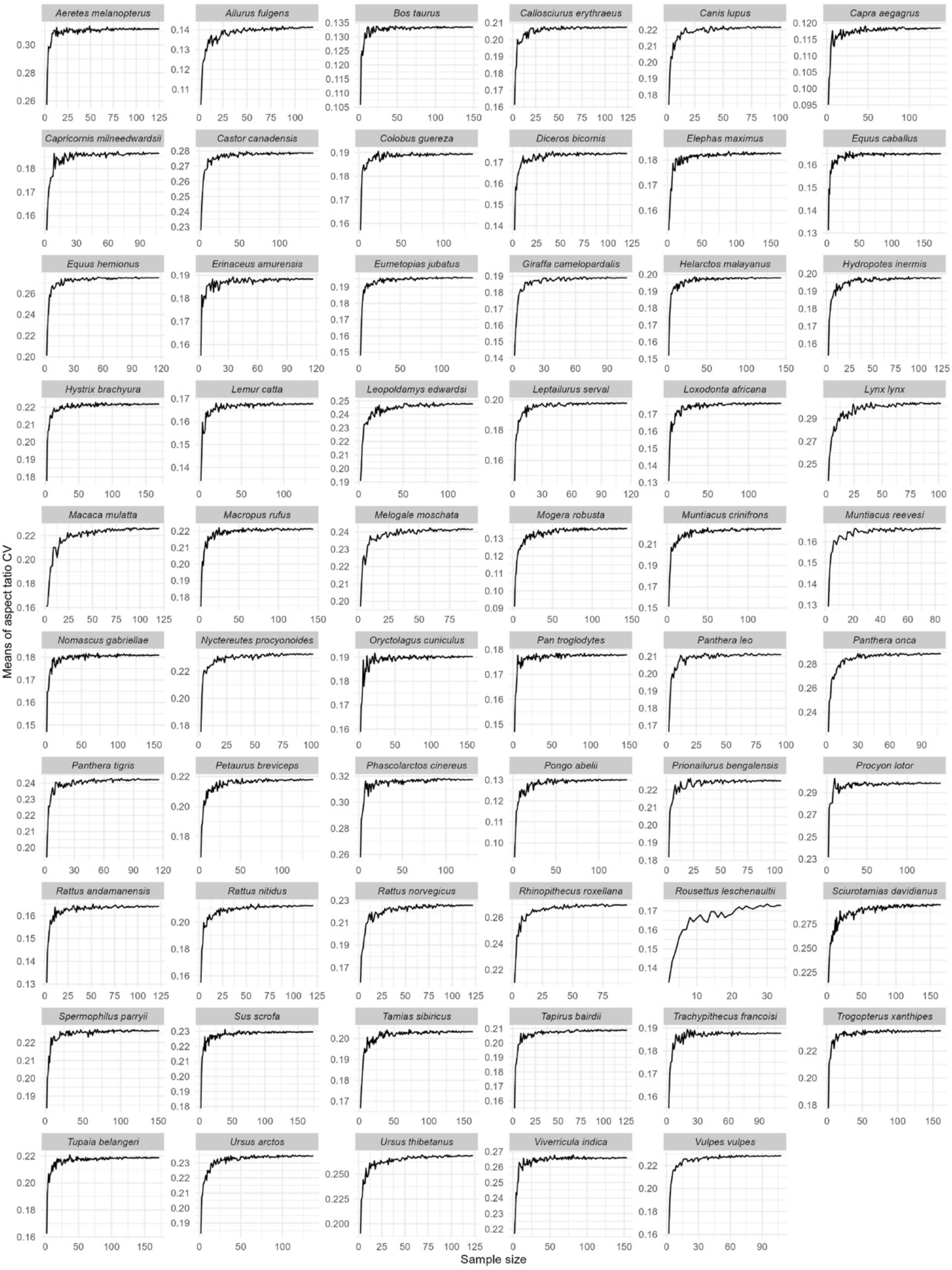

Fig. S10.

The rarefaction curves of sample size to the melanosome aspect ratio CVs.

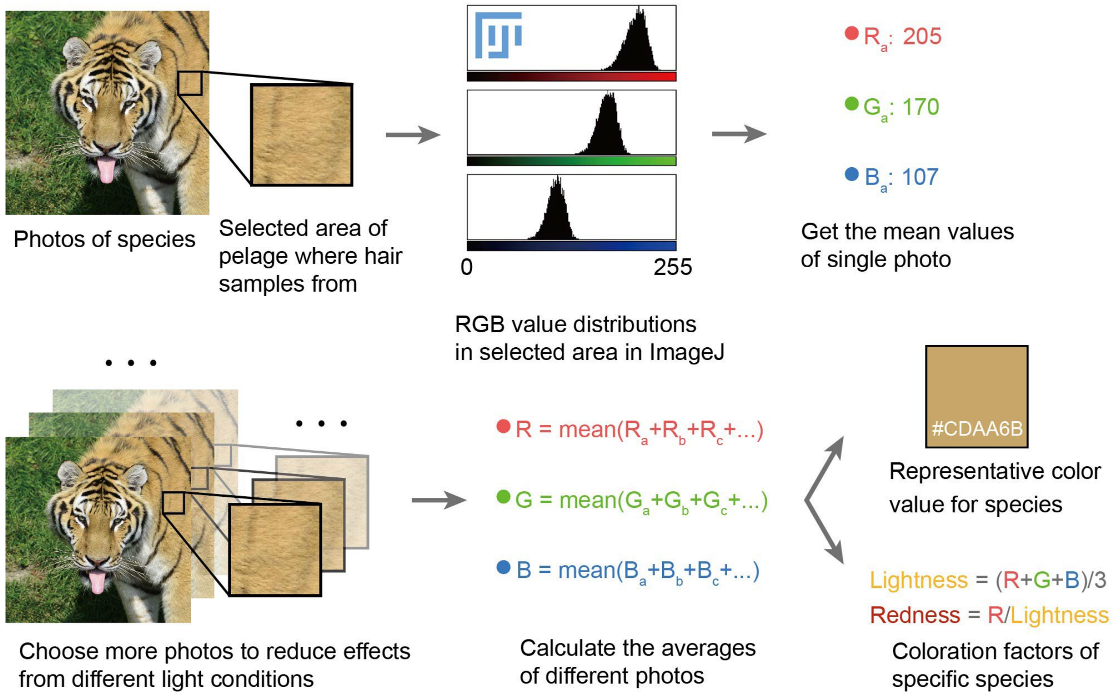

Fig. S11.
The measurement flowchart for pelage color of mammals. Photograph of *Panthera tigris* is from Wikipedia Commons (https://commons.wikimedia.org/wiki/File:Panthera_tigris_altaica_-_Zoo_Sauvage_de_Saint-F%C3%A9licien_-_2016-07-19_(1).jpg). Photo Credit: Antoine Letarte, CC BY 3.0 (https://creativecommons.org/licenses/by/3.0/deed.en). No modifications were made except horizontally flip.

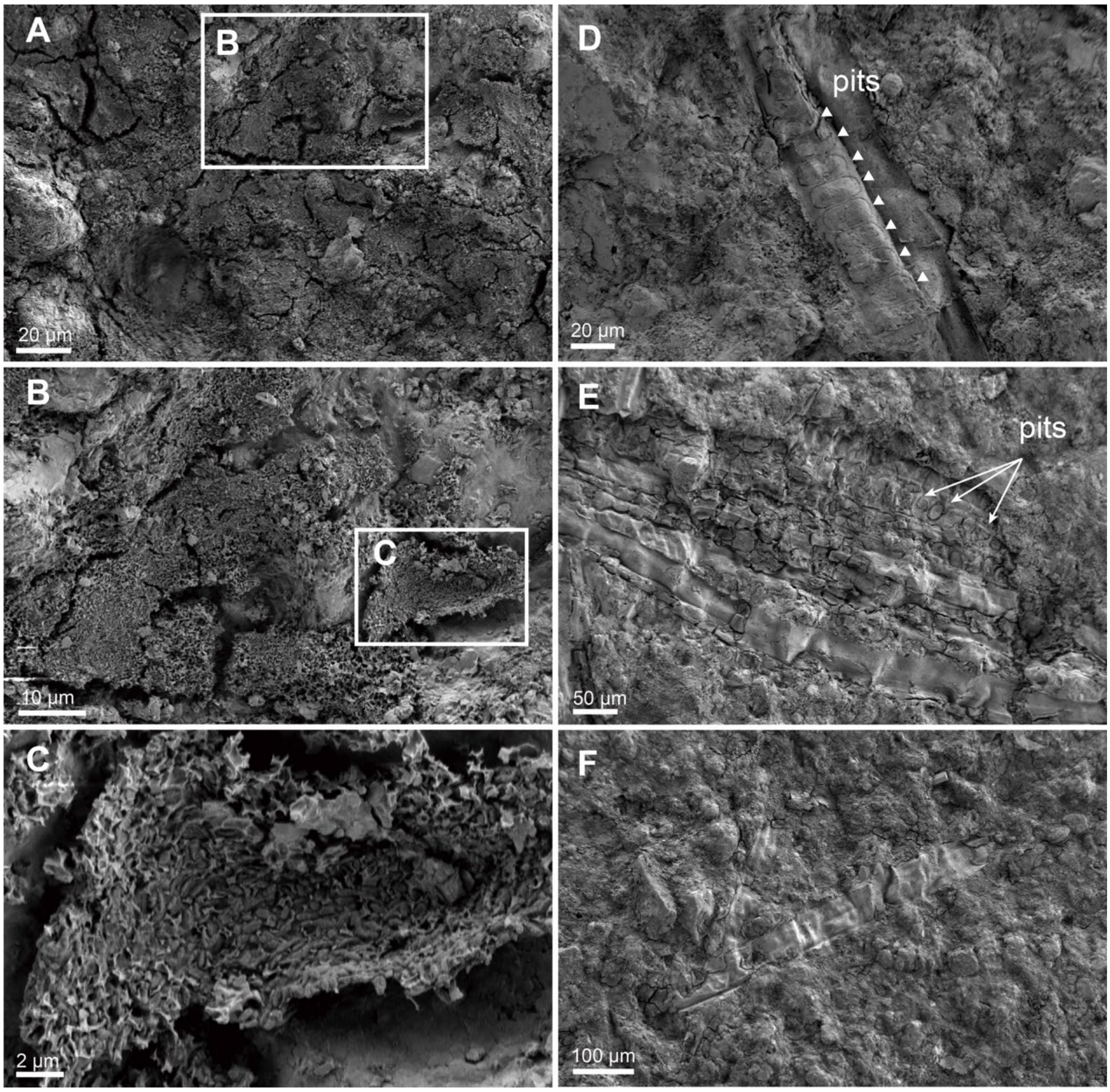

Fig. S12.

The comparison between fossil hair and fossil plant fragments observed in the fossil of *Vilevolodon diplomylos*. (A) to (C) The fossil hair imprint, which has lost the hair keratinized fiber microstructures while melanosomes are surrounded in the fragmented matrix. (D) to (F) Fossil plant fragments, which are indicated by the appearance of pits (D and E) or cuticle (F).

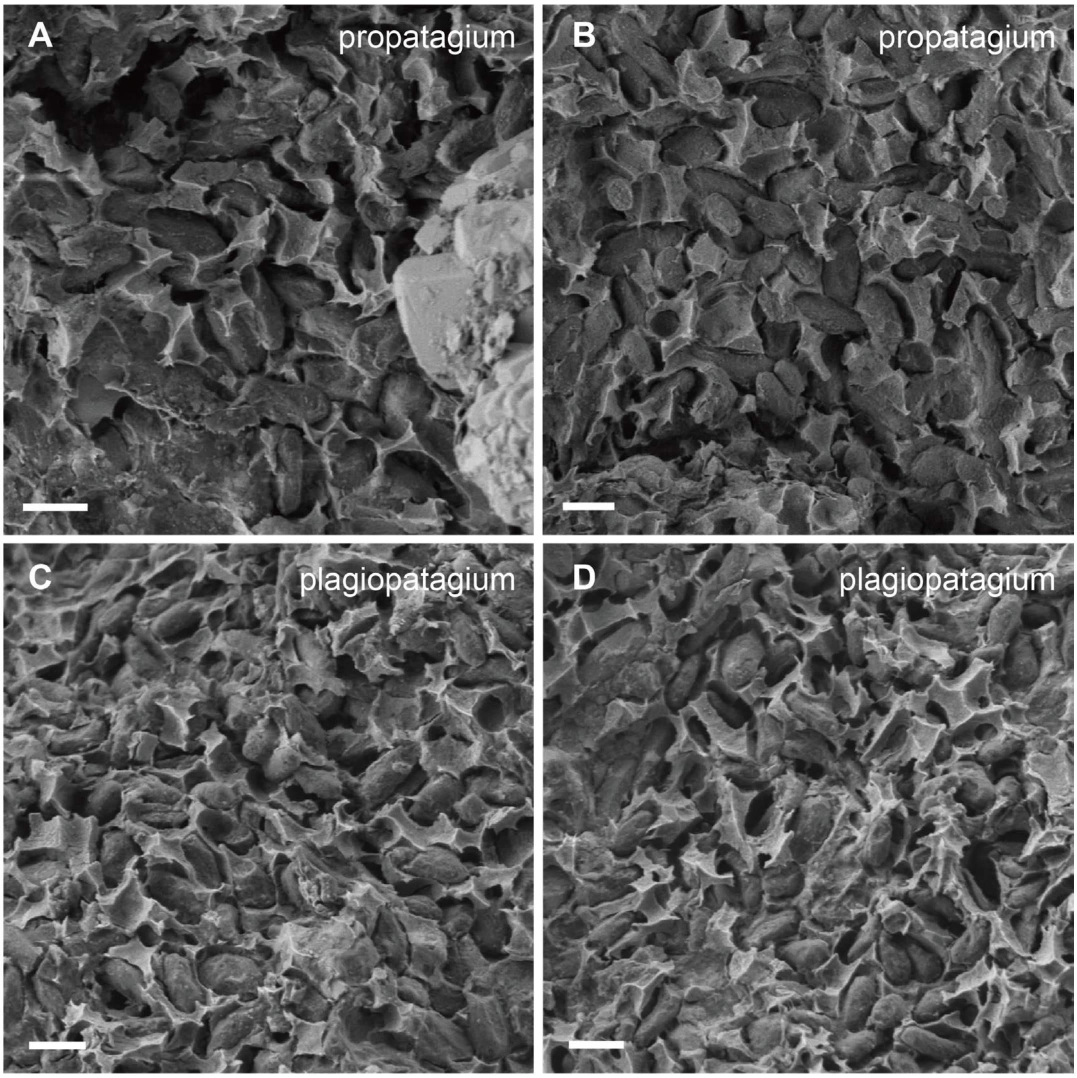

Fig. S13.

The details of melanosomes in the two sampled points on the patagium of *Vilevolodon diplomylos*. The melanosomes of *V. diplomylos* are surrounded by the matrix without order. (A) and (B) show the melanosomes details from propatagium sampled point; (C) and (D) show the melanosomes details from plagiopatagium sampled point. Scale bar: 1 μm.

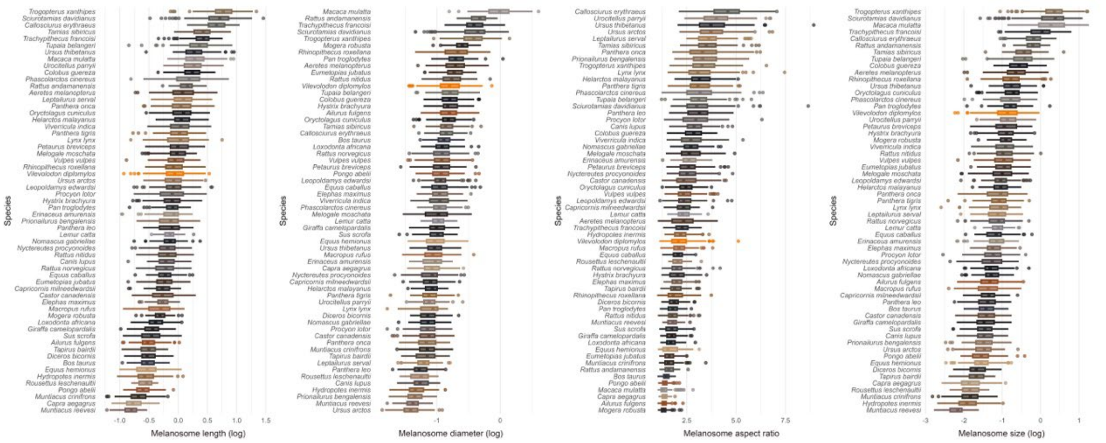

Fig. S14.

Box plots of melanosome geometries. The color indicates the coat coloration of each species and the orange color indicates the fossil sample (Haramiyida: *Vilevolodon diplomylos*).

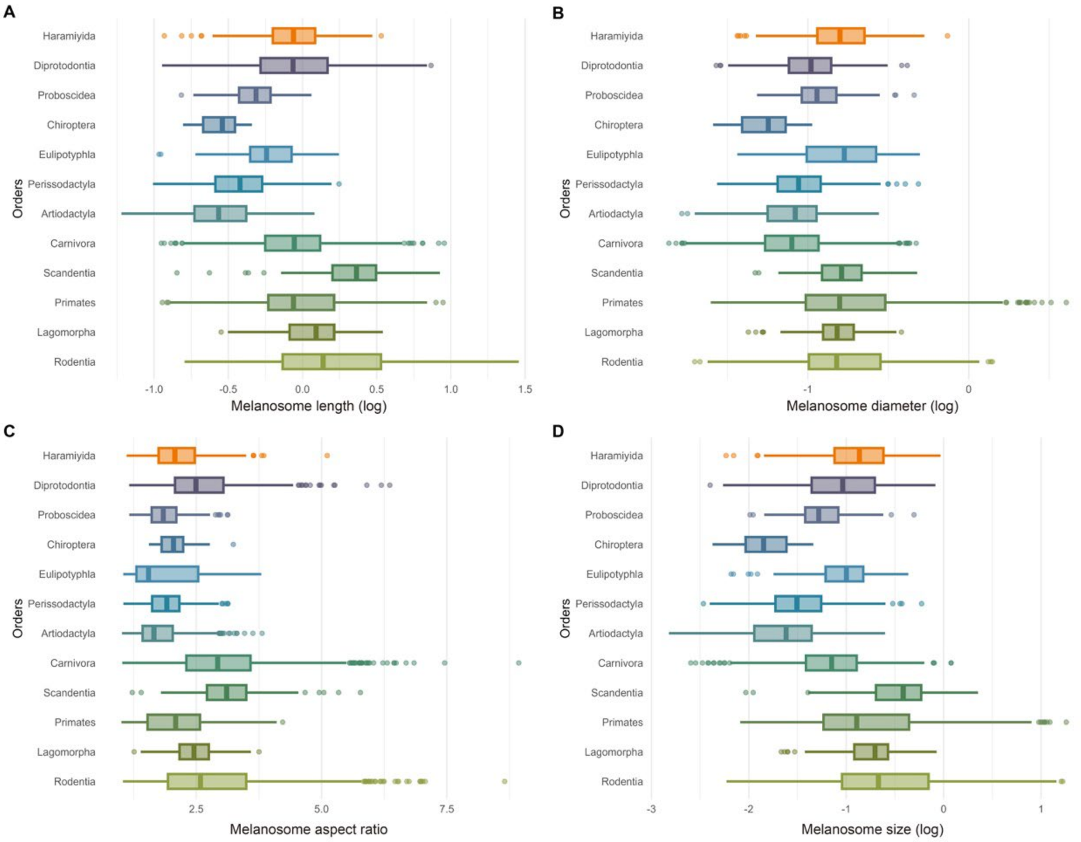

Fig. S15.

Box plots of melanosome geometries across mammalian orders. (A) to (D) are the melanosome length, diameter, aspect ratio and size respectively. The orange color indicates the fossil sample (Haramiyida: *Vilevolodon diplomylos*).

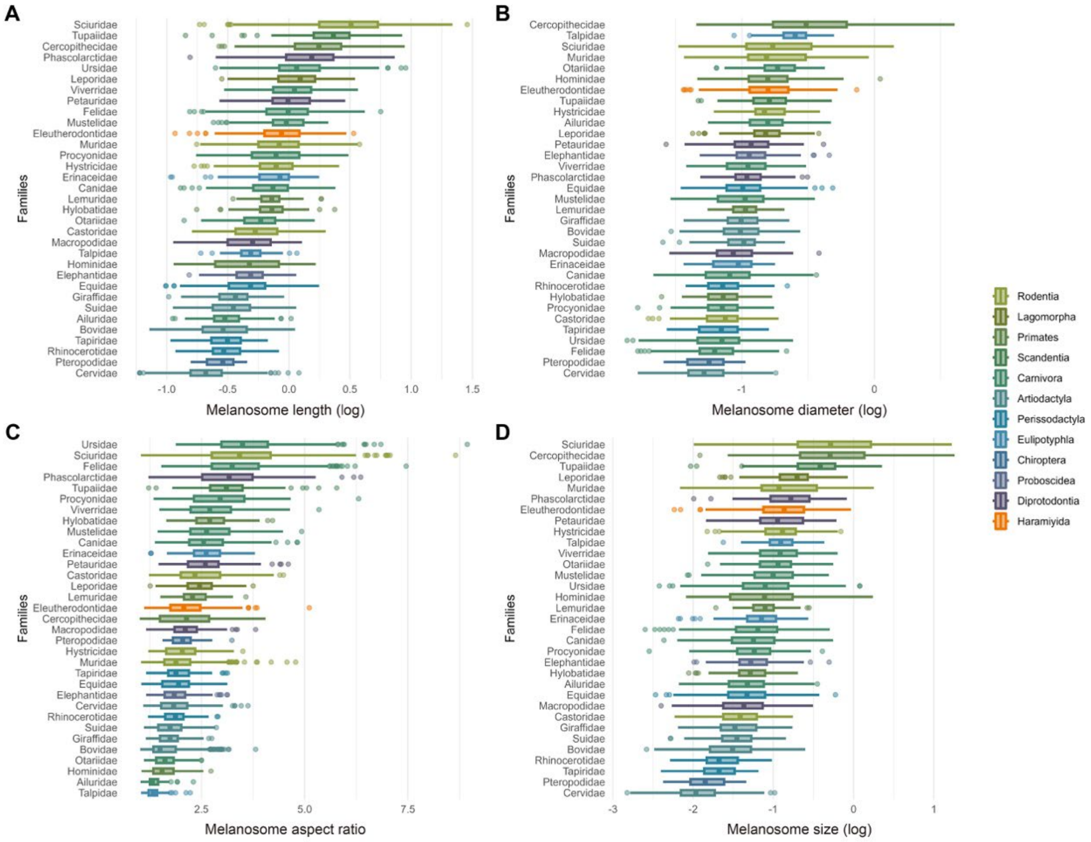

Fig. S16.

Box plots of melanosome geometries across mammalian families. (A) to (D) are the melanosome length, diameter, aspect ratio and size respectively. The orange color indicates the fossil sample (Haramiyida: *Vilevolodon diplomylos*).

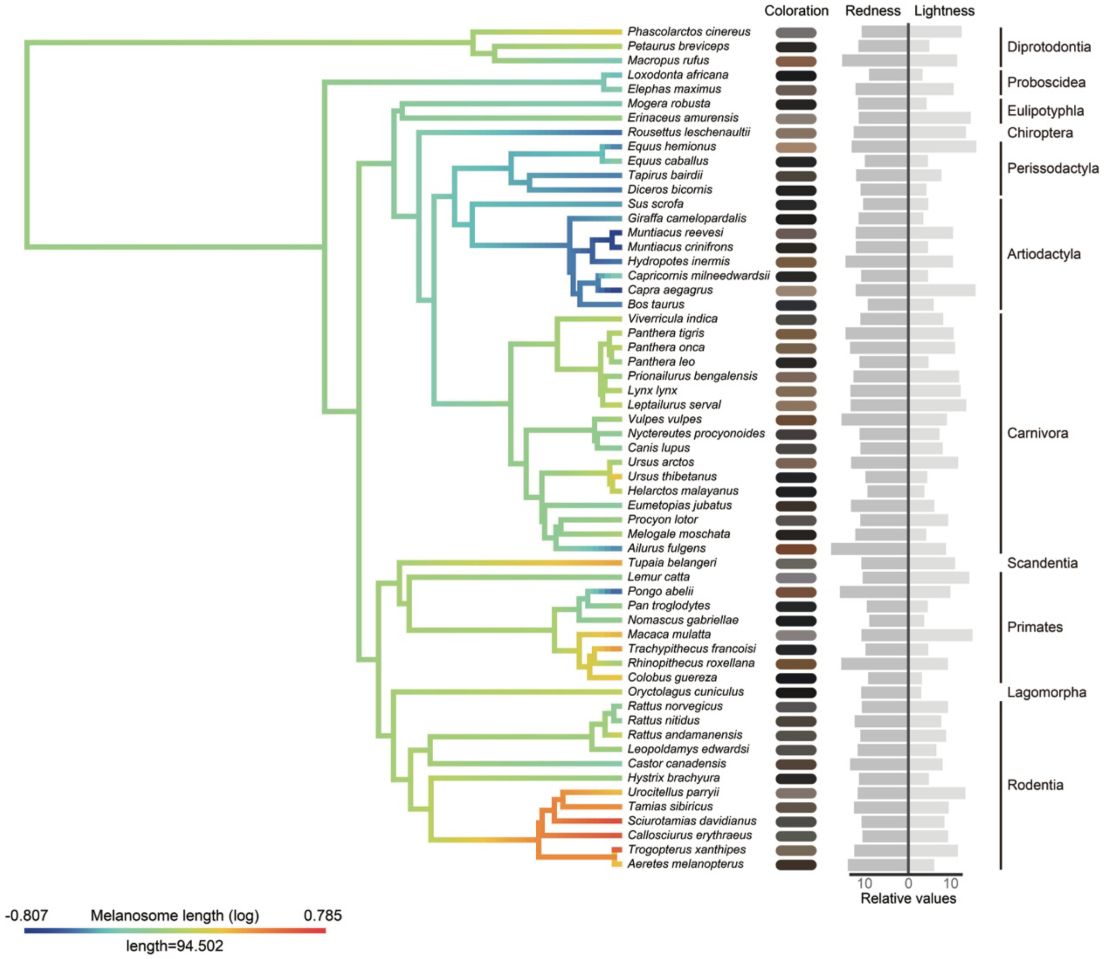

Fig. S17.

Ancestral reconstruction of mammalian melanosome length using Bayesian method.

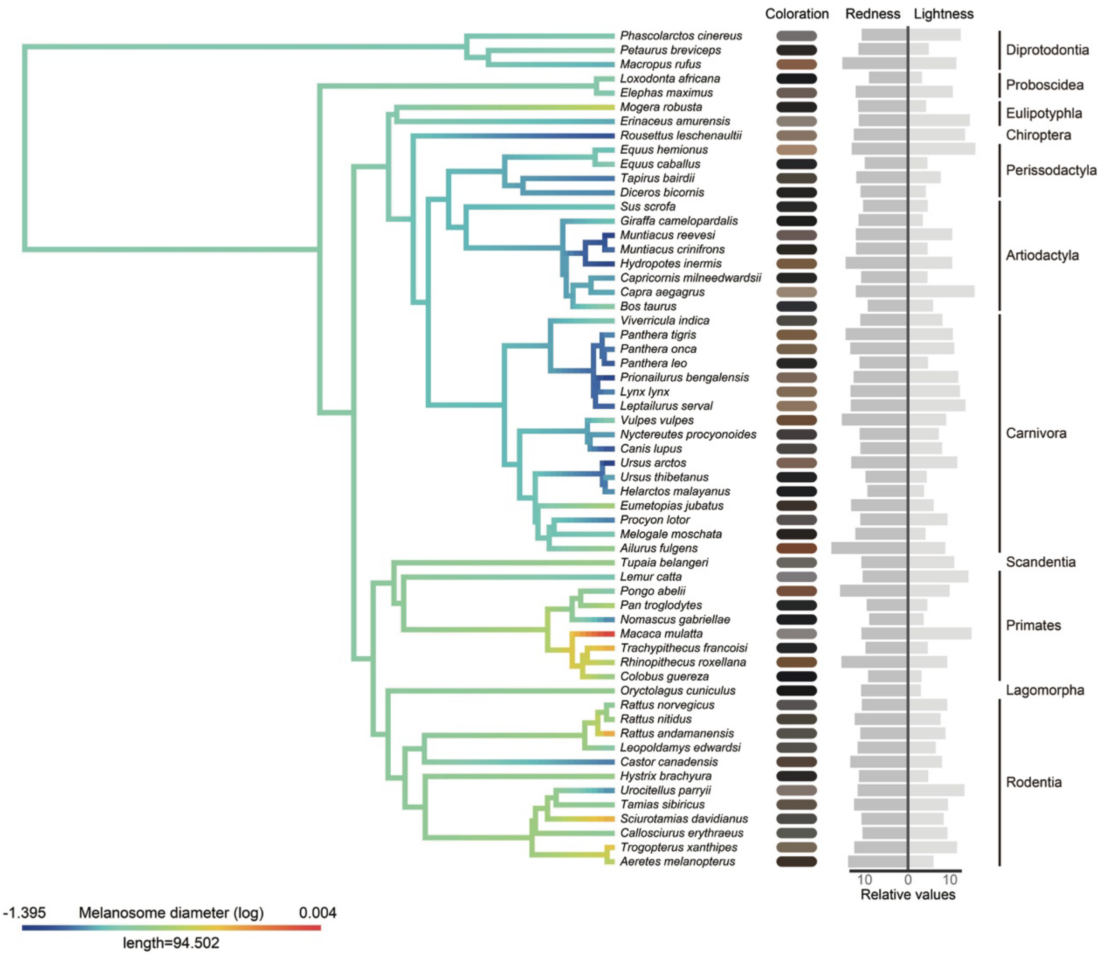

Fig. S18.

Ancestral reconstruction of mammalian melanosome diameter using Bayesian method.

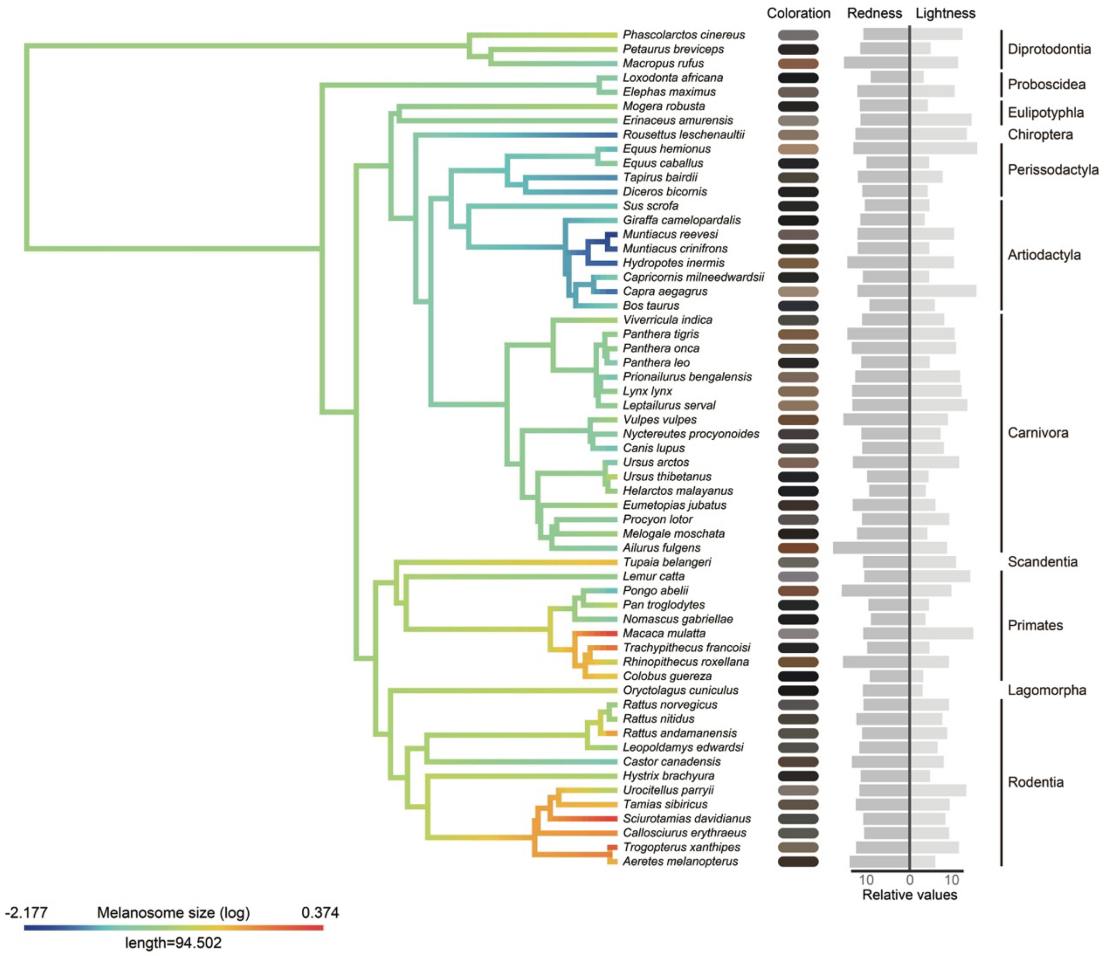

Fig. S19.

Ancestral reconstruction of mammalian melanosome size using Bayesian method.

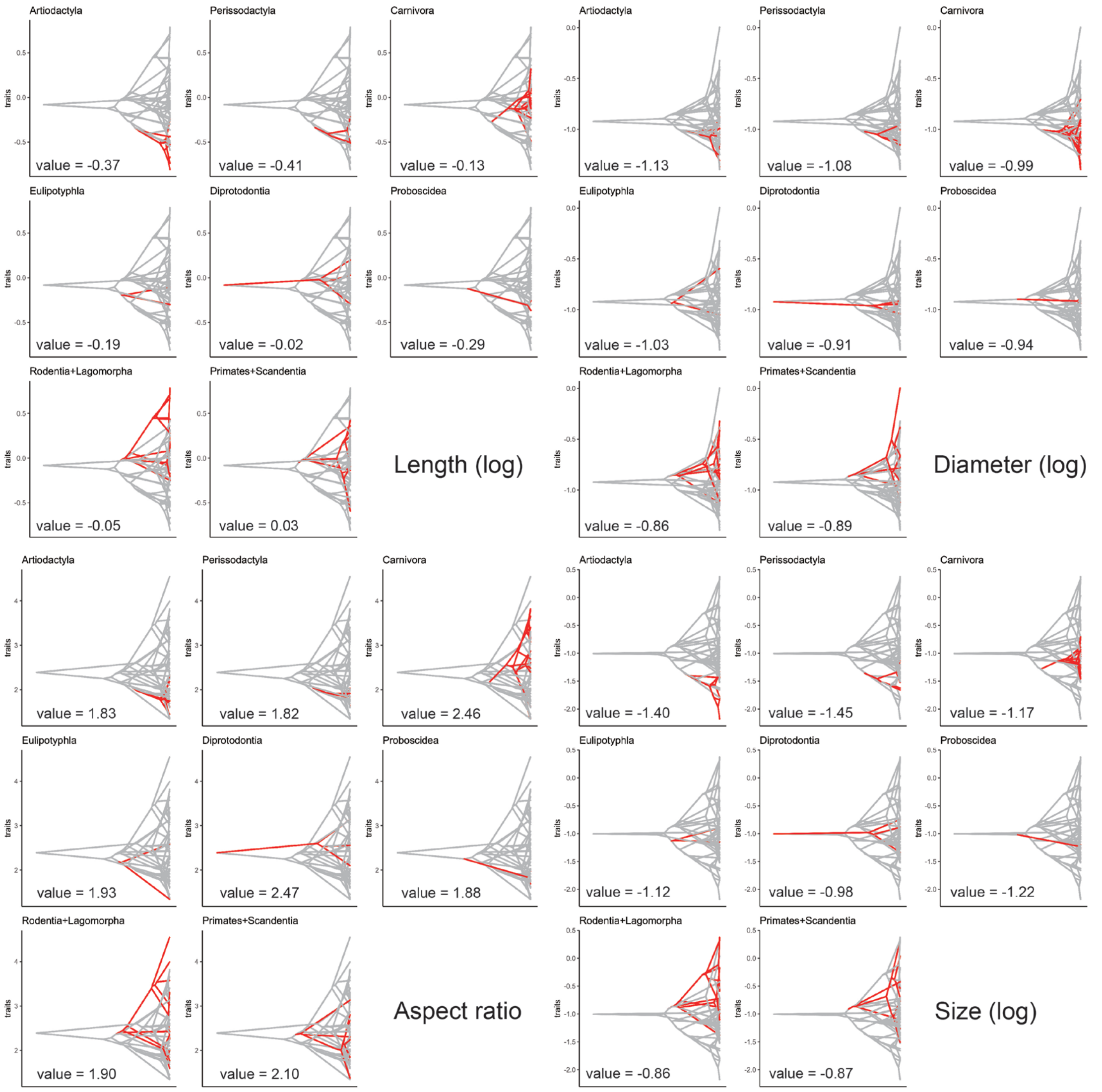

Fig. S20.

The phenograms show the melanosome geometries through time on the summary phylogenetic tree. Values show the estimated states of ancestral nodes of mammalian orders. The Chiroptera is not shown in the images because there is only one sample in our study.

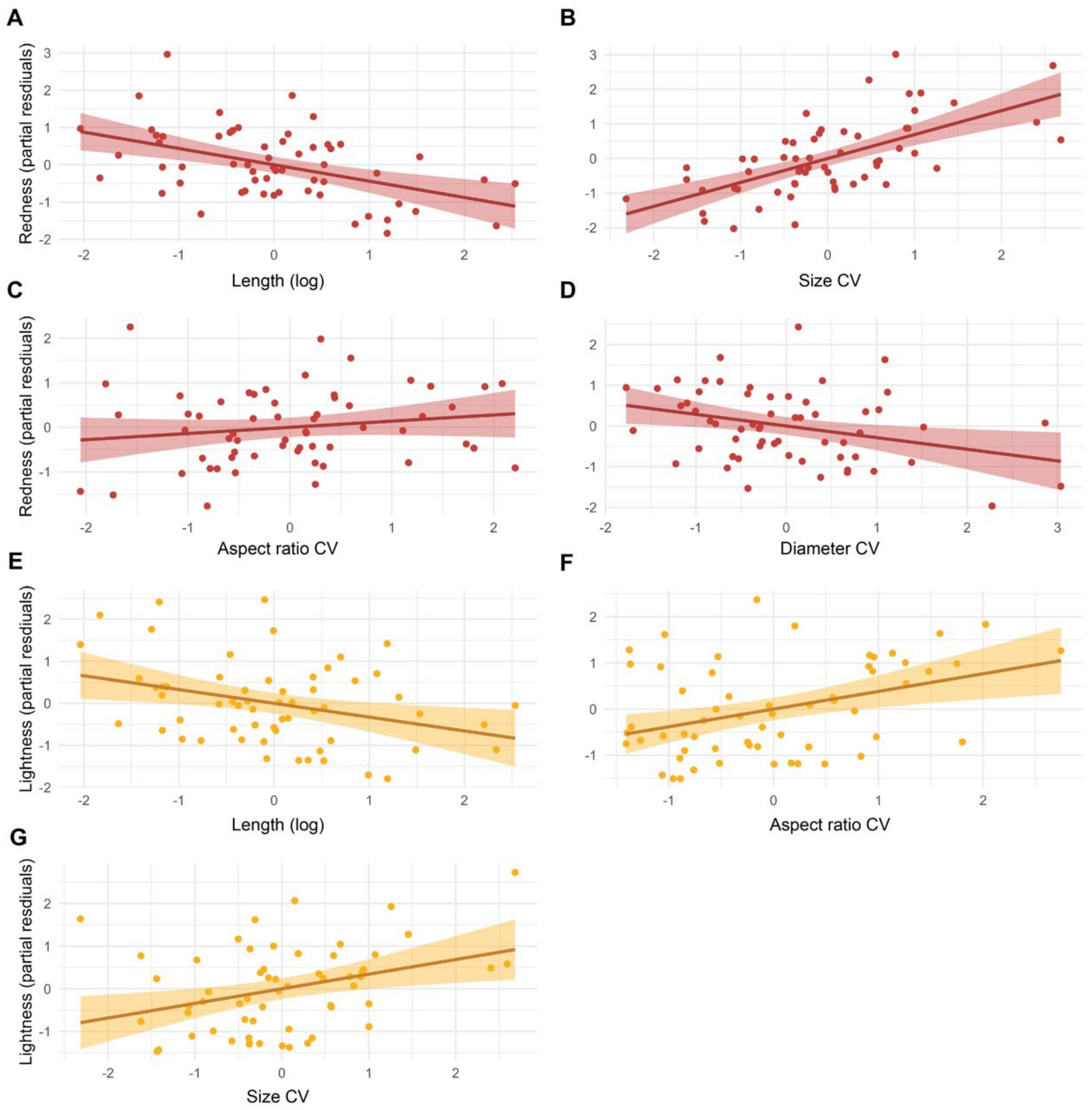

Fig. S21.

The partial residual plots of step-wise multiple variable regressions of melanosome geometries on the coloration parameters. (A) to (D) are the partial residual plots of values of melanosome geometries to the pelage redness in selected area; (E) to (G) are the partial residual plots of melanosome geometries to pelage lightness.

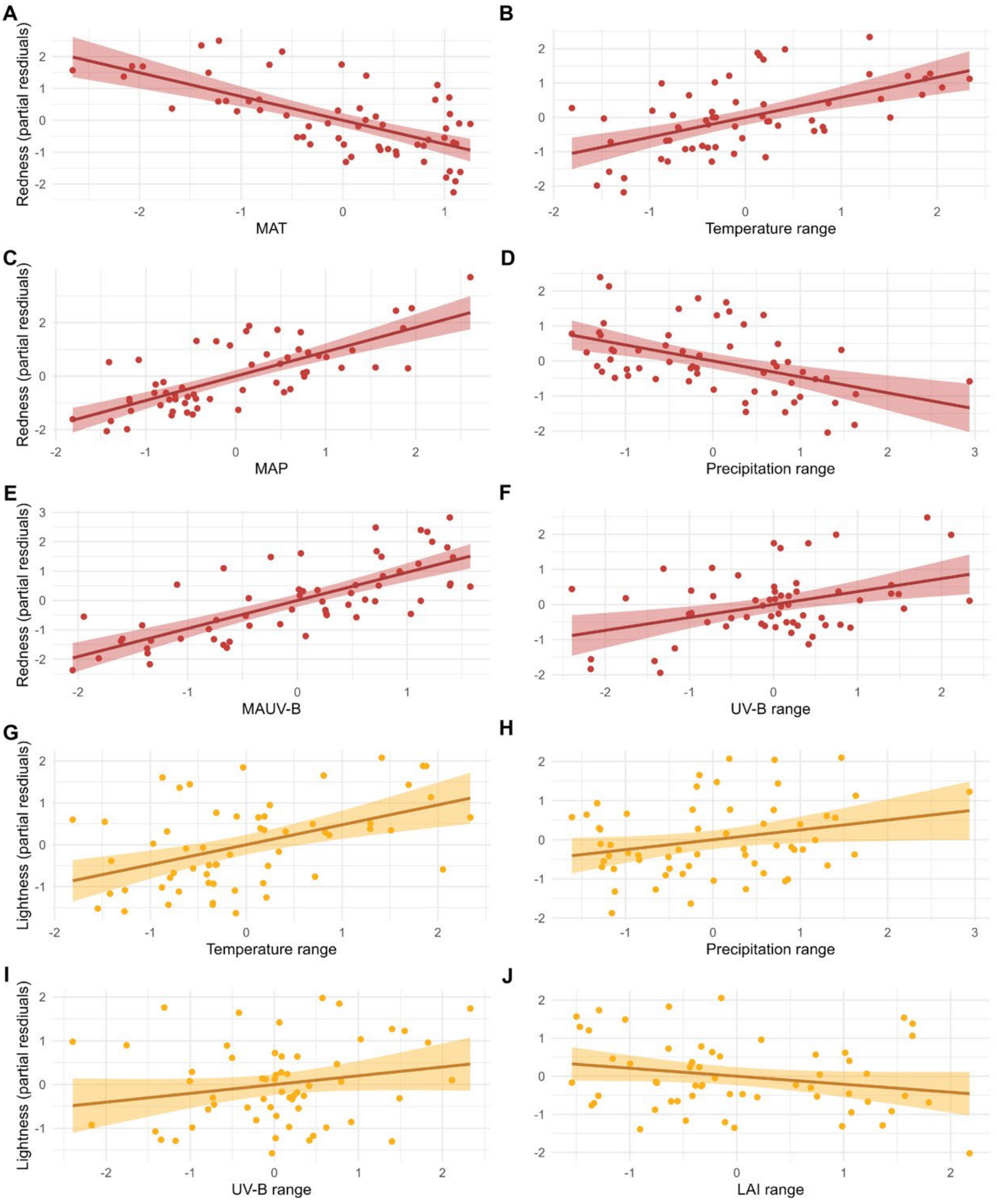

Fig. S22.

The partial residual plots of step-wise multiple variable regressions of ecological and climatic factors on the coloration parameters. (A) to (F) are the partial residual plots of values of melanosome geometries to the pelage redness in selected area; (G) to (J) are the partial residual plots of melanosome geometries to pelage lightness.

Table S1.

List of sources of mammalian hair samples.

Species name	Common name	Sample source	States	*n*
Aeretes melanopterus	Groove-toothed flying squirrel	(1)	Specimen	125
Ailurus fulgens	Red panda	(2)	Extant	119
Bos taurus	Cattle	(3)	Extant	149
Callosciurus erythraeus	Pallas's squirrel	(4)	Extant	124
Canis lupus	Gray wolf	(5)	Specimen	101
Capra aegagrus	Wild goat	(3)	Extant	138
Capricornis milneedwardsii	Chinese Serow	(5)	Specimen	108
Castor canadensis	North American beaver	(6)	Specimen	142
Colobus guereza	Mantled guereza	(2)	Extant	135
Diceros bicornis	Black rhinoceros	(6)	Specimen	120
Elephas maximus	Asian elephant	(6)	Specimen	165
Equus caballus	Horse	(3)	Extant	176
Equus hemionus	Onager	(5)	Specimen	118
Erinaceus amurensis	Amur hedgehog	(7)	Extant	117
Eumetopias jubatus	Steller sea lion	(6)	Specimen	138
Giraffa camelopardalis	Northern giraffe	(2)	Extant	109
Helarctos malayanus	Sun bear	(6)	Specimen	144
Hydropotes inermis	Water deer	(2)	Extant	124
Hystrix brachyura	Malayan porcupine	(8)	Extant	170
Lemur catta	Ring-tailed lemur	(2)	Extant	135
Leopoldamys edwardsi	Edwards's long-tailed giant rat	(4)	Extant	128
Leptailurus serval	Serval	(2)	Extant	116
Loxodonta africana	African elephant	(6)	Specimen	140
Lynx lynx	Eurasian lynx	(2)	Extant	102
Macaca mulatta	Rhesus macaque	(2)	Extant	120
Macropus rufus	Red kangaroo	(2)	Extant	144
Melogale moschata	Chinese ferret-badger	(5)	Specimen	94
Mogera robusta	Ussuri mole	(7)	Extant	144
Muntiacus crinifrons	Hairy-fronted muntjac	(5)	Specimen	141
Muntiacus reevesi	Reeves's muntjac	(5)	Specimen	84
Nomascus gabriellae	Yellow-cheeked gibbon	(2)	Extant	157
Nyctereutes procyonoides	Common raccoon dog	(2)	Extant	102
Oryctolagus cuniculus	European rabbit	(3)	Extant	158
Pan troglodytes	Chimpanzee	(2)	Extant	147
Panthera leo	Lion	(2)	Extant	96
Panthera onca	Jaguar	(2)	Extant	108
Panthera tigris	Tiger	(5)	Specimen	116

Petaurus breviceps	Sugar glider	(3)	Extant	138
Phascolarctos cinereus	Koala	(2)	Extant	131
Pongo abelii	Sumatran orangutan	(2)	Extant	133
Prionailurus bengalensis	Leopard cat	(2)	Extant	106
Procyon lotor	Raccoon	(5)	Specimen	142
Rattus andamanensis	Sikkim rat	(4)	Extant	123
Rattus nitidus	Himalayan field rat	(4)	Extant	121
Rattus norvegicus	Brown rat	(9)	Extant	122
Rhinopithecus roxellana	Golden snub-nosed monkey	(5)	Specimen	95
Rousettus leschenaultii	Leschenault's rousette	(6)	Specimen	34
Sciurotamias davidianus	Père David's rock squirrel	(8)	Extant	165
Sus scrofa	Pig	(3)	Extant	174
Tamias sibiricus	Siberian chipmunk	(6)	Specimen	163
Tapirus bairdii	Baird's tapir	(6)	Specimen	126
Trachypithecus francoisi	François' langur	(5)	Specimen	111
Trogopterus xanthipes	Complex-toothed flying squirrel	(1)	Specimen	161
Tupaia belangeri	Northern treeshrew	(9)	Extant	172
Urocitellus parryii	Arctic ground squirrel	(6)	Specimen	151
Ursus arctos	Brown bear	(6)	Specimen	137
Ursus thibetanus	Asian black bear	(2)	Extant	123
Viverricula indica	Small Indian civet	(2)	Extant	154
Vulpes vulpes	Red fox	(5)	Specimen	108

The parameter n indicates the number of melanosomes measured of each species for analysis. The codes for sample sources correspond to the following institutions: (1) Institute of Zoology, Chinese Academy of Sciences; (2) Nanjing Hongshan Forest Zoo; (3) Nanjing University; (4) Institute of Zoology, Guangdong Academy of Sciences; (5) College of Life Sciences, Nanjing Normal University; (6) Zhejiang Museum of Natural History; (7) Yingliang Stone Natural History Museum; (8) Institute of Vertebrate Paleontology and Paleoanthropology, Chinese Academy of Sciences; (9) Kunming Institute of Zoology, Chinese Academy of Sciences.

Table S2.

Results of step-wise multivariable linear regression models testing the relationship between mammalian coloration and melanosome geometries and distribution.

Coloration	Melanosome parameter	Estimates	Std. Error	*p*-value	Adj. R^2
Redness	Length (log)	-0.438	0.129	**0.001**	**0.250**
	Diameter CV	-0.286	0.209	0.176	
	Aspect ratio CV	0.139	0.134	0.304	
	Size CV	0.692	0.213	**0.002**	
Lightness	Length (log)	-0.328	0.188	0.086	**0.086**
	Aspect ratio	0.383	0.179	**0.037**	
	Size CV	0.343	0.137	**0.016**	

Bold values indicate significant relationships ($p < 0.05$).

Table S3.
The phylogenetic signals of melanosome geometries and coat coloration parameters.

**Variates**	**Pagel's λ**	***p*-value (λ)**
Length (log)	**0.8310**	**0.0000**
Diameter (log)	**0.5380**	**0.0005**
Aspect ratio	**0.9000**	**0.0000**
Size (log)	**0.7340**	**0.0000**
Length CV	**0.6740**	**0.0076**
Diameter CV	0.0001	1.0000
Aspect ratio CV	0.4040	0.1830
Size CV	0.0001	1.0000
Redness	0.0001	1.0000
Lightness	0.0001	1.0000

Pagel's λ values were tested based on the MCC tree, and each parameter was repeated 1,000 times. Bold values indicate significant relationships ($p < 0.05$).

Table S4.

Results of PGLS models testing the relationship between melanosome geometries and environmental variables.

Models	MCC Tree						Average Model (100 trees)			
	**Parameter**	**Estimate**	**Std. Err**	***p*-Value**	**Adj. R^2**	**Pagel's λ**	**Estimate**	**Std. Err**	***p*-Value**	**Pagel's λ**
Length	Mean ann. temperature	0.3727	0.2214	0.0982	0.1073	0.8893	0.3728 (0.3568,0.3912)	0.2217 (0.2168,0.2251)	0.0985 (0.0806,0.1186)	0.8917 (0.8744,0.9087)
	Mean ann. precipitation	-0.3216	0.1565	**0.0448**			-0.3232 (-0.3446,-0.3026)	0.1567 (0.1546,0.1585)	**0.0440** (0.0315,0.0600)	
	Precipitation range	0.4179	0.1669	**0.0153**			0.4183 (0.4014,0.4374)	0.1670 (0.1648,0.1693)	**0.0153 (0.0114,0.0207)**	
	Mean ann. UV-B	-0.3625	0.1910	0.0631			-0.3625 (-0.3796,-0.3454)	0.1912 (0.1868,0.1945)	0.0632 (0.0513,0.0778)	
Diameter	Precipitation range	0.3702	0.1248	**0.0044**	0.1936	0.6240	0.3705 (0.3652,0.3761)	0.1248 (0.1240,0.1258)	**0.0044 (0.0039,0.0051)**	0.6212 (0.5897,0.6859)
	UV-B range	0.3319	0.1147	**0.0054**			0.3325 (0.3269,0.337)	0.1147 (0.1139,0.1157)	**0.0054 (0.0049,0.0063)**	
	Mean ann. LAI	-0.3362	0.1120	**0.0040**			-0.3363 (-0.3403,-0.3303)	0.1120 (0.1110,0.1130)	**0.0040 (0.0037,0.0045)**	
Aspect ratio	Mean ann. precipitation	-0.4778	0.1867	**0.0135**	0.2001	0.9374	-0.4784 (-0.4910,-0.3763)	0.1868 (0.1499,0.1915)	**0.0130 (0.0105,0.0167)**	0.9374 (0.9230,0.9806)
	Precipitation range	0.4162	0.1953	**0.0378**			0.4157 (0.2850,0.4284)	0.1957 (0.1560,0.2009)	**0.0383 (0.0312,0.0464)**	
	Mean ann. UV-B	-0.2231	0.1140	0.0557			-0.2223 (-0.2337,-0.1847)	0.1141 (0.0870,0.1161)	0.0567 (0.0445,0.0713)	
	UV-B range	-0.4225	0.1200	**0.0009**			-0.4223 (-0.4407,-0.4140)	0.1201 (0.1037,0.1218)	**0.0009 (0.0003,0.0011)**	
	Mean ann. LAI	0.1747	0.0862	**0.0478**			0.1753 (0.1406,0.1833)	0.0864 (0.0665,0.0889)	**0.0477** (0.0278,0.0636)	
	LAI range	-0.1526	0.1042	0.1490			-0.1533 (-0.1760,-0.1446)	0.1043 (0.0887,0.1063)	0.1475 (0.0734,0.1695)	
Size	Temperature range	-0.4320	0.2566	0.0981	0.1154	0.8080	-0.4353 (-0.4528,-0.4119)	0.2568 (0.2522,0.2607)	0.0962 (0.0811,0.1124)	0.8068 (0.7862,0.8423)
	Mean ann. precipitation	-0.6026	0.2342	**0.0129**			-0.6050 (-0.6279,-0.5784)	0.2342 (0.232,0.2368)	**0.0125 (0.0103,0.0161)**	
	Precipitation range	0.6909	0.2071	**0.0016**			0.6925 (0.6728,0.7104)	0.2074 (0.2048,0.2097)	**0.0015 (0.0013,0.0019)**	
	Mean ann. UV-B	-0.3549	0.2020	0.0847			-0.3562 (-0.3678,-0.3455)	0.2020 (0.1976,0.2058)	0.0840 (0.0759,0.0906)	
	Mean ann. LAI	-0.2137	0.1023	**0.0415**			-0.2144 (-0.2178,-0.2068)	0.1024 (0.1003,0.1043)	**0.0414 (0.0371,0.0469)**	

The results of PGLS models based on the MCC tree are on the left side and the results of model averaging based on the 100 trees block are on the right. The numbers in the parentheses indicate 95% CI of model average and the bold values indicate significant relationships ($p < 0.05$). Although some average *p*-values are lower than 0.05, the 95% confidence intervals could cross this threshold, indicating uncertainty in the phylogeny arose in our study.

Table S5.

Results of BayesTraits models testing the relationship between melanosome geometries and environmental variables.

	**BayesTraits regression estimation (1000 Trees)**			
	**Parameter**	**Lower 95%**	**Lower 95%**	**pMCMC**
Length	Mean ann. temperature	-0.067	0.834	0.195
	Mean ann. precipitation	-0.620	0.022	0.060
	Precipitation range	0.062	0.773	**0.019**
	Mean ann. UV-B	-0.724	0.095	0.106
Diameter	Precipitation range	0.128	0.637	**0.005**
	UV-B range	0.110	0.575	**0.007**
	Mean ann. LAI	-0.590	-0.135	**0.002**
Aspect ratio	Mean ann. precipitation	-0.883	-0.079	**0.020**
	Precipitation range	0.002	0.851	**0.048**
	Mean ann. UV-B	-0.462	0.025	0.060
	UV-B range	-0.647	-0.130	**0.003**
	Mean ann. LAI	-0.025	0.370	0.069
	LAI range	-0.406	0.040	0.123
Size	Temperature range	-0.925	0.121	0.114
	Mean ann. precipitation	-1.088	-0.109	**0.015**
	Precipitation range	0.280	1.174	**0.005**
	Mean ann. UV-B	-0.755	0.063	0.105
	Mean ann. LAI	-0.451	-0.006	0.050

Bold values indicate significant relationships ($p < 0.05$).

Table S6.
Results of BPMM models testing the relationship between melanosome geometries and environmental variables

Bayesian phylogenetic mixed model (BPMM, 100 Trees)				
	**Parameter**	**Lower 95%**	**Lower 95%**	**pMCMC**
Length	Mean ann. temperature	-0.103	0.826	0.124
	Mean ann. precipitation	-0.640	0.001	0.051
	Precipitation range	0.078	0.758	**0.017**
	Mean ann. UV-B	-0.753	0.041	0.076
Diameter	Precipitation range	0.124	0.624	**0.005**
	UV-B range	0.097	0.560	**0.006**
	Mean ann. LAI	-0.562	-0.113	**0.004**
Aspect ratio	Mean ann. precipitation	-0.864	-0.099	**0.015**
	Precipitation range	0.011	0.819	**0.044**
	Mean ann. UV-B	-0.461	0.009	0.060
	UV-B range	-0.667	-0.170	**0.001**
	Mean ann. LAI	-0.004	0.357	0.055
	LAI range	-0.373	0.057	0.149
Size	Temperature range	-0.959	0.092	0.103
	Mean ann. precipitation	-1.079	-0.125	**0.015**
	Precipitation range	0.268	1.110	**0.002**
	Mean ann. UV-B	-0.768	0.054	0.087
	Mean ann. LAI	-0.421	-0.007	**0.043**

Bold values indicate significant relationships ($p < 0.05$).

Table S7.
Results of step-wise multivariable linear regression models testing the relationship between mammalian coloration and environmental variables.

**Coloration**	**Ecological factors**	**Estimates**	**Std. Error**	***p*-value**
Redness	Mean ann. temperature	-0.748	0.310	**0.019**
	Temperature range	0.582	0.364	0.116
	Mean ann. Precipitation	0.909	0.331	**0.008**
	Precipitation range	-0.456	0.252	0.076
	Mean ann. UV-B	0.956	0.295	**0.002**
	UV-B range	0.368	0.149	**0.017**
Lightness	Temperature range	0.476	0.188	**0.014**
	Precipitation range	0.252	0.176	0.158
	UV-B range	0.201	0.143	0.165
	LAI range	-0.210	0.136	0.129

Bold values indicate significant relationships ($p < 0.05$).

Data S1. (separate file)
Fossil_Melanosome_Data.csv

Data S2. (separate file)
Extant mammalian melanosome geometry and habitat environmental data.xlsx

Data S3. (separate file)
Extant_Melanosome_Data.csv
